# Zoledronic Acid-Loaded Hybrid Hyaluronic Acid/Polyethylene Glycol/Nano-Hydroxyapatite Nanoparticle: Novel Fabrication and Safety Verification

**DOI:** 10.3389/fbioe.2021.629928

**Published:** 2021-02-15

**Authors:** Yan Xu, Zheng Zhang, Hehui Wang, Wu Zhong, Chengmei Sun, Wei Sun, Hongwei Wu

**Affiliations:** ^1^Department of Thoracic Medicine Oncolog, Hunan Cancer Hospital and The Affiliated Cancer Hospital of Xiangya School of Medicine, Central South University, Changsha, China; ^2^Zhejiang University-University of Edinburgh Institute, School of Medicine, Zhejiang University, Haining, China; ^3^Department of Orthopedics, Ningbo Yinzhou Second Hospital, Ningbo, China; ^4^Department of Orthopedics, Hunan Cancer Hospital and The Affiliated Cancer Hospital of Xiangya School of Medicine, Central South University, Changsha, China

**Keywords:** hybrid, nanoparticle, drug release, cytotoxicity, immune response, zoledronic acid

## Abstract

Osteosarcoma is a malignant tumor that often occurs in adolescents and children. Zoledronic acid, a new-generation bisphosphonate, has been widely used as an antitumor drug to inhibit bone metastasis. However, the rapid renal elimination results in low effective concentrations. Meanwhile, high-dose intravenous zoledronic acid administration leads to severe side effects. The present study fabricated an organic–inorganic hybrid nanoparticle as the carrier of zoledronic acid. The rod-like nanoparticle, which had 150-nm length and 40-nm cross-sectional diameter, consisted of a hyaluronic acid/polyethylene glycol (HA-PEG) polymer shell and a nano-hydroxyapatite (nHA) core, with zoledronic acid molecules loading on the surface of nHA and clearance of HA-PEG shell. The nanoparticle was characterized by microscopic analysis, *in vitro* release study, cytotoxicity analysis, and *in vivo* immune response examination. Results showed that the compact and stable structure could achieve high drug loading efficiency, sustained drug release, and great biocompatibility. *In vitro* and *in vivo* experiments revealed the low cytotoxicity and acceptable immune response under low-dose nanoparticle treatment, indicating its potential application for future osteosarcoma therapeutic strategies.

## Introduction

Osteosarcoma is the most common bone malignancy, which usually occurs in adolescents and children (Moore and Luu, 2014). Since it mostly occurs at the distal femur, proximal tibia, and humerus, it seriously affects the patient’s movement and survival (Bielack et al., 2002). Therapeutic strategies for osteosarcoma include surgery and chemotherapy, which are based on high dose antitumor agent uptake, such as methotrexate (Luetke et al., 2014).

Bisphosphonates have been proved to inhibit bone resorption, which could be applied in tumor-induced osteolysis treatment (Van Poznak et al., 2011). Zoledronic acid (ZOL), a new-generation bisphosphonate, is widely used to inhibit osteosarcoma growth and bone metastasis (Poirier et al., 2003; Lipton, 2008). As reported, ZOL could suppress cancer cell growth by inducing cellular apoptosis and cell cycle arrest (Chang et al., 2012). Other studies also revealed effects including osteoclastogenesis inhibition, angiogenesis inhibition, T cell activation, and metastasis inhibition (Dass and Choong, 2007; Muraro et al., 2007; Koto et al., 2009; Tsubaki et al., 2012; Ouyang et al., 2018). However, ZOL concentration decreases rapidly after intravenous injection, with <10% of C_max_ after 4 h and <1% of C_max_ after 24 h (Novartis Pharmaceuticals Corporation, 2016). In cancer patients, about 39% ZOL is excreted renally, while the remaining part may accumulate at bone because of its high bone affinity (Novartis Pharmaceuticals Corporation, 2016). The low effective concentration and rapid elimination give rise to high-dose ZOL treatment, along with severe side effects including renal impairment and pyrexia (Black et al., 2007; Novartis Pharmaceuticals Corporation, 2016). Hence, nanoparticle-targeted drug delivery is proposed as a potential method to achieve ZOL-mediated osteosarcoma therapy with low dose and high efficiency.

Nanomedicine has provided new sights for antitumor drug delivery and targeting. Advantages using nanoparticles (NPs) as the drug carrier include the following: (1) protection of the drug from degradation and elimination; (2) increase in the circulation and retention time; (3) higher drug loading efficiency and solubility; and (4) accurate targeting with ligand modification (Gu et al., 2013). For example, Federman et al. (2012) fabricated an anti-ALCAM-hybrid-polymerized liposomal NP which targets osteosarcoma-associated cell surface antigen ALCAM (Activated Leukocyte Cell Adhesion Molecule). Loading with chemotherapy drug doxorubicin (DXR), the hybrid NP, showed enhanced cytotoxicity to osteosarcoma cells compared to untargeted NP (Federman et al., 2012). However, most studies only showed *in vitro* cytotoxicity analysis, while the *in vivo* safety and immune response triggered by hybrid micro-particles was infrequently investigated.

In this study, we constructed a zoledronic acid-loaded hybrid hyaluronic acid/polyethylene glycol/nano-hydroxyapatite nanoparticle (HA-PEG-nHA-ZOL NP). Since HA is the ligand of CD44, a highly expressed cancer stem cell marker on the osteosarcoma cell surface, the organic shell, HA-PEG polymer, could play a role in osteosarcoma targeting and sustained drug release (Morath et al., 2016; Liu et al., 2018). PEG can perform pH-sensitive shedding, which promotes drug release in an acidic solid tumor environment (Li et al., 2019). The inorganic core, nHA, on account of its good biocompatibility and bioactivity, is commonly used as a bone repair material and drug carrier (Venkatesan and Kim, 2014), especially for antitumor drugs (Dai et al., 2015; Sun et al., 2017; Pandey et al., 2018). However, most of the articles in micro/nanoparticles focused on cytotoxicity to tumorigenic cells, while the toxicity to normal cells and tissues was often neglected. Our study provided a systemic and comprehensive evaluation on the safety of ZOL-loaded nanoparticles. In traditional ZOL therapy, 0.1 mg/kg was considered as a high-dose administration, while in our *in vivo* study, 100 μl, 250 μg/ml of NPs was considered as a moderate equivalent dosage, which is five times the regular dose (Wolfe et al., 2011). In general, this study aimed to synthesize and characterize of a new hybrid nanoparticle, along with cytotoxicity test and organ tolerance evaluation. Additionally, immune response after NP intravenous injection was evaluated, aiming to prove the safety of NP injection under a proper dose manner.

## Materials and Methods

### Reagents

Low molecular weight (5,000–10,000 dalton) hyaluronic acid (HA) was acquired from Shandong Freda Biotechnology (Shandong, China). Dimethyl sulfoxide, deuterium oxide, and DMSO were acquired from Sinopharm Chemical Reagent (China). Dialysis bags (7,000 kDa and 8,000–14,000 kDa) were acquired from Shanghai Yuanye Bio-Technology (Shanghai, China). Polyethylene glycol (PEG, 8,000 dalton), tetrabutylammonium hydroxide (TBA, 40% in H2O), and *N,N’*-dicyclohexylcarbodiimide (DCC) were acquired from Aladdin Reagent (Shanghai, China). Dulbecco’s modified Eagle’s medium/high modified (DMEM-H), phosphate buffer saline (PBS), antibiotic–antimycotic, and fetal bovine serum (10099-141) were acquired from Gibco (United States). Red Blood Cell Lysis Buffer (C3702) and Cell counting kit-8 were acquired from Beyotime Biotechnology (Shanghai, China). Sodium zoledronate was acquired from Solarbio (Beijing, China). Nano hydroxyapatite was acquired from Aladdin Reagent (Shanghai, China). CD68-PE, CD3-FITC, and CD8-APC antibodies were acquired from BioLegend (United States). LIVE/DEAD Cell Imaging Kit was acquired from Dojindo (Japan). CD68 and CD3 antibodies were acquired from Santa-Cruz (United States). Anti-goat and anti-rabbit secondary antibodies were acquired from Bosterbio (United States). Alanine aminotransferase ALT assay kit (C052), aspartate aminotransferase AST assay kit (C072), alkaline phosphatase assay kit (C003), blood urea nitrogen determination kit (C010), and creatinine assay kit (C074) were acquired from Changchun Huili Biotech (Changchun, China).

### Cells and Animals

A total of 293T cells were acquired from ATCC and cultured in DMEM supplemented with 10% fetal bovine serum (FBS), 1% penicillin and streptomycin at 37°C, and 5% CO_2_ (Heal Force, China). A culture dish, 24-well plates, and 96-well plates were acquired from Corning. C57BL/6 mice (6–8 weeks) were acquired from Hunan SJA Laboratory Animal Co., Ltd (Hunan, China). All mice were sacrificed by 1% pentobarbital sodium injection (80 mg/kg). The animal protocol was approved by the Institutional Animal Care and Use Committee (IACUC) of Hunan Cancer Hospital (KYJJ-2016-009).

### Synthesis of HA-PEG-nHA-ZOL Nanoparticles

#### Synthesis of HA-TBA

HA-TBA was prepared according to the methods in Oh et al. (2009). A total of 1 g of HA was weighed into a small beaker, kept stirred, and dissolved in 20 mL of deionized water. After being completely dissolved, 1 mL of TBA solution was added and stirred at room temperature for half an hour. The solution was frozen at −20°C and then lyophilized in a vacuum freeze-drying agent to obtain an HA-TBA polymer.

#### Synthesis of HA-PEG-nHA

PEG-nHA was prepared according to the methods in Yang et al. (2020). A total of 1 g of HA-TBA, 0.5 g of PEG, and 0.5 g of nHA were weighed into a small flask, which contained 30 mL of DMSO liquid. The liquid was then sonicated in an ice bath using an ultrasonic disperser (Langee, Shenzhen, China) for 60 min. After that, 0.5 g of DCC was added into the liquid. The container was transferred to a 50°C oil bath and stirred for 2 days. After 2 days, the reaction liquid was added to a 50-mL centrifuge tube and centrifuged at 5,000 rpm (Eppendorf Centrifuge 5810 R, Germany) for 20 min. The supernatant was removed, and the pellet was dispersed in 10 mL of DMSO using an ultrasonic disperser. The above suspension was added to a dialysis bag (8,000–14,000 kDa), and the deionized water was changed every half an hour. After 12 h, the liquid in the dialysis bag was taken out and lyophilized to obtain HA-PEG-nHA material.

#### Preparation of Blank and Drug-Loaded NPs

A total of 10 mg of HA-PEG-nHA material was weighed and added into 10 mL of PBS, then sonicated for 1 min to obtain HA-PEG-nHA NPs.

Zoledronic acid-loaded nanoparticles were prepared by using an adsorption method. A total of 0.5 g of HA-PEG-nHA material and 0.2 g of sodium zoledronate were added into 10 mL of deionized water. After 1 min of ultrasonic dispersion treatment, the above liquid was stirred for 4 h, then centrifuged at 5,000 rpm for 20 min, and the precipitate was lyophilized (Biobase, Shandong, China) to obtain HA-PEG-nHA-ZOL NPs powder. A total of 10 mg of HA-PEG-nHA-ZOL material was weighed and added to 10 mL of PBS, then sonicated for 1 min to obtain HA-PEG-nHA-ZOL NPs.

### Morphological Analysis of NPs

Transmission electron microscopy (TEM) was used to perform morphological analysis of NPs. HA-PEG-nHA-ZOL NPs with 2% phosphorous acid stained were dropped on a copper mesh coated with a carbon support film. After drying, the morphology was observed under a transmission electron microscope.

### Size Distribution and Stability of NPs

Dynamic light scattering (DLS) was used to measure the size distribution and stability of NPs. The prepared HA-PEG-nHA NP and HA-PEG-nHA-ZOL NP nanoparticles were put into a cuvette, and we put the cuvette into a dynamic light scattering particle size analyzer for testing. Each sample tested for 3 times, 1 min each time. The test conditions were as follows: argon ion laser, wavelength 658 nm, temperature 25 ± 0.1°C, and dynamic light scattering angle 90 degrees. The zeta potential was simultaneously measured; the operating conditions are 11.4 v/cm, 13.0 mA, and 25°C; and the sample solvent was diluted with distilled water. DLS was measured for three times and the average calculated.

### Encapsulation and Loading Efficiency (FTIR and NMR)

A small amount of solid nHA, HA-TBA, and HA-PEG-nHA NP samples was taken out and mixed with dried potassium bromide powder, after being grinded and pressed into a transparent sample potassium bromide tablet, and we measured the infrared spectrum of the sample.

HA-PEG-nHA-ZOL NPs and 10 mg each of ZOL were taken and dispersed in 1 ml of deuterated heavy water, and a nuclear magnetic resonance proton spectrometer was used to calculate the drug loading of HA-PEG-nHA-ZOL NPs according to the following formula:

$$\text{Ai}={\text{A}}_{\delta \,7.26}+A_{\delta \,7.41}$$

$$\text{DS}=\frac{{\text{A}}_{\mathrm{Zol}}}{{\text{A}}_{\mathrm{NPs}}}\times 100\%$$

Where *i* = ZOL or HA-PEG-nHA -ZOL NPs, A_δ 7_._26_ and A_δ 7_._41_ are both a characteristic hydrogen peak on the imidazole group. Measured NMR for three times and calculate the average.

### *In vitro* Drug Release Assay

A concentration-absorbance standard curve was established for ZOL phosphoric acid at 210 nm. A total of 20 mg of HA-PEG-nHA -ZOL NPs was accurately weighed and dispersed in 10 mL of deionized water under an ice bath. Then, this liquid was placed in a dialysis bag (10,000 kDa), and the dialysis bag was placed in a PBS/HCl–PBS buffer solution with pH of 7.4 and 5.8, respectively, and protected from light at 37°C and 100 rpm. Within a certain time, the entire release medium is replaced with fresh medium, and the old medium is retained. The ultraviolet-visible spectrophotometer was used to measure the ZOL concentration in old media, and the release rate was calculated according to the following formula:

$$\text{Q}\%=\left(\sum_{t=0}^{t}{\text{M}}_{t}\right)/\left({\text{m}}_{\mathrm{NP}}\times \text{LC}\%\right)$$

Where *t* = 0, 0.5, 1, 2, 4, 8, 12, 16, 24, 36, 48, 60, 72 h and M_*t*_ is the product of the old medium volume and the concentration.

### Cytotoxicity Assay

Cell viability was tested by the cell counting kit-8 (CCK-8). A total of 8,000 of 293T cells were seeded into 96-well plates, each absorbance group for 4 wells. The groups are listed in Table 1. Notably, NPs that had the concentrations lower than 2.5 μg/ml were considered as “low-dose” for cells; NPs that had the concentrations higher than 5 μg/ml were considered as “high-dose” for cells.

**TABLE 1:** Drug concentrations for CCK-8.

Cell	Drug	Concentration
293T	PBS	/
293T	NPs	0.625 μg/ml, 1.25 μg/ml, 2.5 μg/ml, 5 μg/ml, 10 μg/ml
293T	NPB	0.625 μg/ml, 1.25 μg/ml, 2.5 μg/ml, 5 μg/ml, 10 μg/ml
293T	nHA	0.625 μg/ml, 1.25 μg/ml, 2.5 μg/ml, 5 μg/ml, 10 μg/ml
293T	ZOL	0.25 μg/ml, 0.5 μg/ml, 1 μg/ml, 2 μg/ml, 4 μg/ml

One day later, drugs were added into the wells and culture for 2 days, and 10 μl CCK-8 solution was added into each well and cultured for 3 h. A microplate reader (Tecan, Spark, Switzerland) was used to measure the absorbance at 450 nm, and the cell viability was calculated according to the following formula:

$$viability\;rate\;\left(\%\right)\text{=}\frac{OD\;(Experimental\;Group)-OD\;(Blank\;group)}{OD\;(Control\;group)-OD\;(Blank\;group)}\times 100\%$$

### Live/Dead Dyeing

A total of 293T cells were cultured in a 24-well plate. After culturing for 1 day, the cells were treated with drugs, each group having 2 wells. Groups are listed in Table 2.

**TABLE 2:** Drug concentrations for Live/Dead Dyeing.

Cell	Drug	Concentration
293T	PBS	/
293T	NPs	2.5 μg/ml, 10 μg/ml
293T	NPB	1.25 μg/ml, 2.5 μg/ml, 5 μg/ml, 10 μg/ml
293T	nHA	1.25 μg/ml, 2.5 μg/ml, 5 μg/ml, 10 μg/ml
293T	ZOL	1 μg/ml, 4 μg/ml

After a 2-day culture, a 200-μl culture medium which contains Calcein-AM (CAM) and PI (1:500 diluted) was added to each well of 293T cells. After 30 min of incubation, the cells were observed under a fluorescence microscope (Leica DMi8, Germany).

### Nanoparticle *in vivo* Injection

Nanoparticles were caudal intravenously injected into mice. The concentrations and doses of NPs used in the current study are listed in Table 3.

**TABLE 3:** In vivo drug delivery concentrations and doses.

Result	Concentration	Dose
H&E staining	125 μg/ml, 250 μg/ml, 500 μg/ml, 1000 μg/ml	100 μl
Liver and kidney function	250 μg/ml, 500 μg/ml, 1000 μg/ml	100 μl
IHC staining	0 μg/ml, 500 μg/ml, 1000 μg/ml	100 μl
Peripheral blood flow cytometry analysis	250 μg/ml	100 μl

### H&E Staining

Healthy mice were treated with NPs in different concentrations (details in Table 3). Notably, NPs that had concentrations lower than 250 μg/ml were considered as “low-dose” for mice; NPs that had concentrations higher than 500 μg/ml were considered as “high-dose” for mice.

After 3 days, mice were sacrificed and organs including the kidney, lung, heart, liver, and brain were fixed with 4% paraformaldehyde overnight. The organs were then dehydrated and embedded in paraffin. The organs were then sectioned into 5-μm slices and stained with hematoxylin and eosin (H&E). The stained sections were imaged under an inverted phase-contrast microscope (Olympus CX23, Japan).

### Immunohistochemistry Staining

The lung, liver, spleen, and kidney were collected and embedded in paraffin 3 days after injection. After sectioning into 5-μm slices, CD3 and CD68 immunohistochemistry (IHC) staining was performed. For IHC staining, dewaxing and rehydration of paraffin sections were first treated with 3% hydrogen peroxide in methanol solution for 15 min. After PBS flush, the sections were blocked with 10% secondary antibody serum for 30 min. Diluted primary antibodies (1:100) were added and incubated overnight at 4°C. After PBS flush, the secondary antibody was added and incubated for 30 min at room temperature. Then, diaminobenzidine (DAB) coloration was performed and hematoxylin was used to restain nuclei. Dehydration and covering of the slide were performed.

### Liver and Kidney Function

Liver function was evaluated according to alanine aminotransferase (ALT), aspartate aminotransferase (AST), and alkaline phosphatase (ALP) level. Peripheral blood was collected from the caudal vein. After 10 min of 4000-rpm centrifuge, supernatant was collected and ALT, AST, and ALP assay was performed. Serum ALT, AST, and ALP levels were determined by an automatic biochemical analyzer Chemray 240 (Rayto Life and Analytical Sciences, United States).

Kidney function was evaluated according to blood urea nitrogen (BUN) and creatinine (CR) level. Peripheral blood was collected from the caudal vein. After 10 min of 4000-rpm centrifuge, supernatant was collected and BUN and creatinine assay was performed. Serum BUN and creatinine levels were determined by an Automatic biochemical analyzer Chemray 240.

### Flow Cytometry Analysis

Peripheral blood was collected 3 days after drug delivery. RBC lysis buffer (5 ml) was added into 1 ml blood, lysed for 5 min, and centrifuged at 450 *g* and 4°C for 5 min (Eppendorf Centrifuge 5424 R, Germany). The supernatant was discarded, resuspended with 1 ml ice-cold PBS buffer, filtered through a cell strainer (70 μm), and centrifuged at 450 *g* and 4°C for 15 min. The supernatant was discarded and resuspended with 100 μl ice-cold PBS buffer. CD68-PE, CD3-FITC, and CD8-APC antibodies (1:100) were added and incubated at 4°C for 30 min and centrifuged at 450 *g* and 4°C for 5 min. The supernatant was discarded and resuspended with 200 μl ice-cold PBS buffer. Novo Express was used to perform flow cytometry analysis.

### Statistical Analysis

After the normality test, data subjected to normal distribution were analyzed by one-way-ANOVA followed by multiple comparisons between groups using Tukey’s *post hoc* test. A Mann–Whitney *U* test was used for analyzing flow cytometry data. All statistical analysis was carried out with GraphPad Prism 8.0 software. A *p*-value of < 0.05 is considered significant.

## Results

### Synthesis and Characterization of HA-PEG-nHA-ZOL Nanoparticles

**FIGURE 1:**
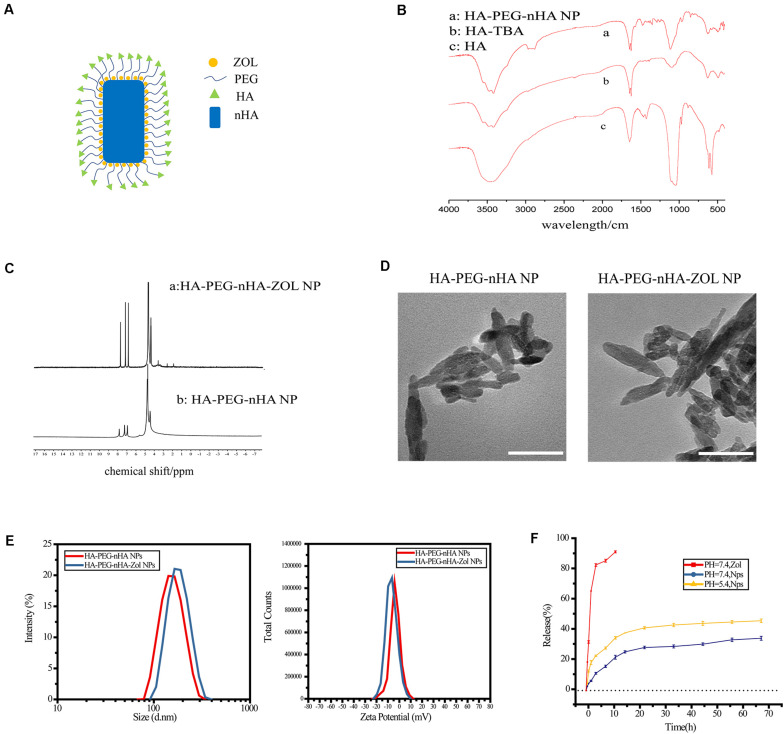
Synthesis and characterization of HA-PEG-nHA-ZOL NPs. **(A)** Schematic of HA-PEG-nHA-ZOL nanoparticles. **(B)** Synthetic routes revealed by FTIR on (a) HA-PEG-nHA NP, (b) HA-TBA, and (c) HA-PEG-nHA nanoparticles. **(C)** Drug loading efficiency revealed by NMR on (a) HA-PEG-nHA-ZOL NP and (b) HA-PEG-nHA NP. **(D)** TEM figure of HA-PEG-nHA NP (left) and HA-PEG-nHA-ZOL NP (right). Scale bar, 100 nm. **(E)** Size distribution (left) and zeta potential (right) of HA-PEG-nHA NP and HA-PEG-nHA-ZOL NP. **(F)** Drug release of NPs in the neutral environment (pH = 7.4) and acidic environment (pH = 5.4). Free zoledronic acid released in the neural environment (pH = 7.4) was considered as control.

Briefly, the synthesis of HA-PEG-nHA-ZOL nanoparticles consisted of three steps. First, HA and TBA solutions were mixed, frozen, and vacuum freeze-dried to obtain the HA-TBA polymer. PEG and nHA were subsequently added into HA-TBA and ultrasonic dispersed to acquire HA-PEG-nHA nanoparticles. Finally, with ZOL drug loading, HA-PEG-nHA-ZOL nanoparticles were synthesized. The encapsulation process was checked by Fourier transform infrared spectrometry (FTIR), revealing the synthetic routes from HA to HA-TBA and finally HA-PEG-nHA (Figure 1B). The drug loading process and efficacy were measured by nuclear magnetic resonance (NMR). In Figures 1C.a,C.b, the peak at 4.65 ppm refers to the methylene peak on zoledronic acid sodium. In Figure 1C.a, peaks at 3 to 4 ppm refer to the methine peaks on HA and PEG, peaks at 1.9 to 2.6 ppm refer to the hydroxyl peaks on HA, and peaks at 7 to 8 ppm are characteristic peaks of hydrogen on the imidazole ring. According to the peak areas of the above peaks, free zoledronic sodium was used as the external standard, and the ZOL loading efficiency could be calculated, which was 41.3 ± 2.31%. Compared with traditional polymeric nanoparticles, the hybrid nanoparticle in this study had a much higher drug loading rate, which might be related to the high affinity between ZOL and nHA.

As shown in the schematic of the HA-PEG-nHA-ZOL nanoparticle, the hybrid nanoparticle was constructed by a nHA core recruiting hydrophilic ZOL molecules, wrapping with a PEG-HA polymer shell (Figure 1A). Transmission electron microscopy (TEM) images of blank and ZOL-loaded nanoparticles exhibited a rod-like geometrical profile, with about 150 nm length and 40 nm cross-sectional diameter (Figure 1D). Diameters of blank and ZOL-loaded nanoparticles were precisely measured by dynamic light scattering (DLS). As shown in Table 1 and Figures 1D,E, the average diameter of HA-PEG-nHA NPs was 159.0 ± 2.3 nm. When loaded with ZOL, the average diameter was 182.5 ± 1.7 nm. The structure and stability of NPs were analyzed according to the zeta potential. As shown in Table 4 and Figure 1E, the zeta potential of HA-PEG-nHA was −6.01 ± 0.23 mV while that of HA-PEG-nHA-ZOL was −7.03 ± 0.19 mV. The electronegativity of ZOL reduced the surface potential of nanoparticles after drug loading, which efficiently stabilized the structure.

**TABLE 4:** Sizes and zeta potentials of blank and drug loaded NPs.

NPs	Size (d.nm)	Zeta potential (mV)
HA-PEG-nHA NPs	159.0 ± 2.3	−6.01 ± 0.23
HA-PEG-nHA-Zol NPs	182.5 ± 1.7	−7.03 ± 0.19s

Ultraviolet spectrophotometry was used to measure the drug release rate *in vitro*. As shown in Figure 1F, dissociative ZOL displayed a rapid release rate with 91.43% liberated drug within 12 h. Relatively, HA-PEG-nHA-ZOL NPs exhibited a slower and constant drug release rate. At pH of 5.4 and 7.4, HA-PEG-nHA-ZOL NPs were released 30.08 and 20.54% ZOL, respectively, within 12 h, 64.11%, and 49.62% ZOL, respectively, within 72 h. The results above indicated that controlled and sustained drug release could be realized using HA-PEG-nHA-ZOL NPs, maintaining the long-lasting therapeutic effect with a single injection. Moreover, the higher drug release rate in an acidic environment indicates that HA-PEG-nHA-ZOL NPs could be an acid-sensitive carrier to promote the drug release in acidic solid tumor sites or ischemic regions.

### HA-PEG-nHA-ZOL NPs Exhibited Good Biocompatibility *in vitro*

**FIGURE 2:**
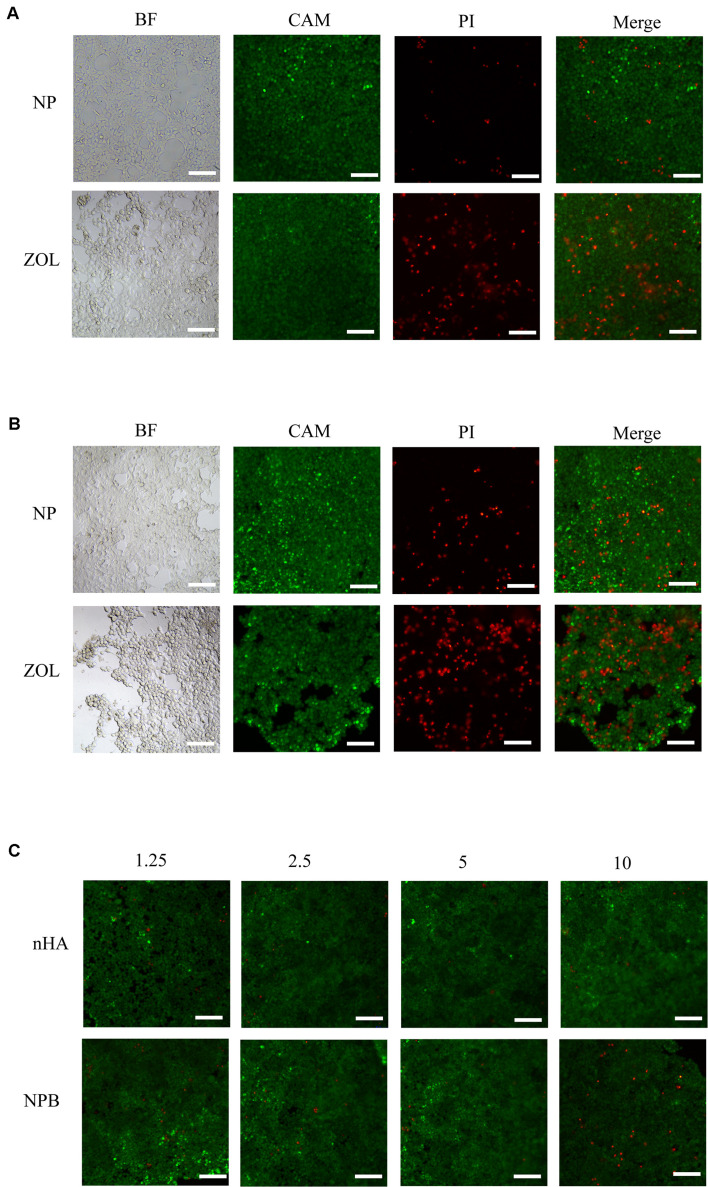
Live/dead cell dyeing. Live/dead cell dyeing was performed on 293T cells treated with **(A)** intermediate concentration (2.5 μg/ml) of NP and ZOL, **(B)** high concentration (10 μg/ml) of NP and ZOL, **(C)** gradient concentration (1.25 μg/ml, 2.5 μg/ml, 5 μg/ml, 10 μg/ml) of nHA and NPB. BF, bright field; CAM, Calcein-AM; PI, Propidium Iodide; scale bar, 100 μm.

**FIGURE 3:**
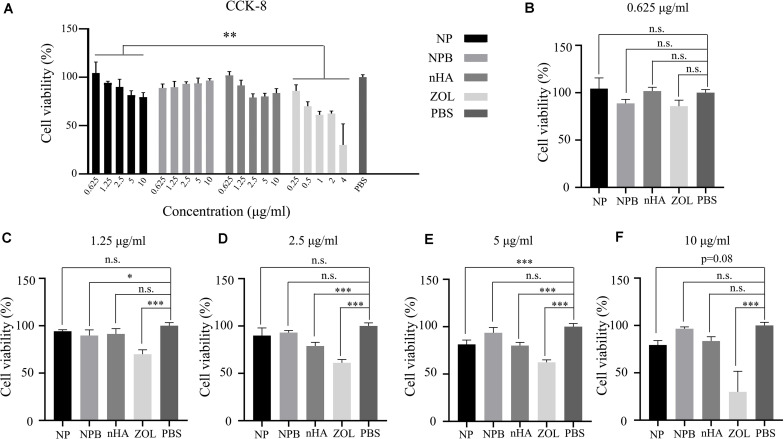
CCK-8 assay. **(A)** Relative cell viability after gradient concentration of NP, NPB, nHA, and ZOL treatment. PBS was used as the control group. Relative cell viability after NP, NPB, nHA, and ZOL treatment with concentrations of **(B)** 0.625 μg/ml (NP, NPB, nHA), 0.25 μg/ml (ZOL); **(C)** 1.25 μg/ml (NP, NPB, nHA), 0.5 μg/ml (ZOL); **(D)** 2.5 μg/ml (NP, NPB, nHA), 1 μg/ml (ZOL); **(E)** 5 μg/ml (NP, NPB, nHA), 2 μg/ml (ZOL); **(F)** 10 μg/ml (NP, NPB, nHA), 4 μg/ml (ZOL). One-way-ANOVA, ^∗^*p* < 0.05; ^∗∗^*p* < 0.01; ^∗∗∗^*p* < 0.005; n.s., non-significance.

Cell live/dead dyeing and cell counting kit-8 (CCK-8) assay were performed to evaluate the cytotoxicity of NPs to 293T cells. A total of 293T cells were treated with gradient concentration of NPs and ZOL (details in Table 2), and cell viability was measured after 48 h. To achieve equivalent drug dose control, the NPs and ZOL concentrations were converted based on the 40% drug loading rate. With low-dose NP (<2.5 μg/ml) treatment, 293T cells showed high viability. When treated with a low dose of free ZOL (the same dose was loaded in NPs, < 1 μg/ml), 293T cells showed lower viability and poor cell condition (Figure 2A). However, under high-dose NPs (>5 μg/ml) and ZOL (>2 μg/ml) treatment, 293T cells exhibited low cell viability (Figures 2B, 3A–F). We propose that even with sustained drug release, high-dose NPs had toxic effect due to high ZOL concentration in culture medium after 48 h of drug release.

To examine the cytotoxicity of the HA-PEG-nHA NP itself, we performed both cell live/dead dyeing and CCK-8 assay 48 h after treating 293T cells with gradient concentrations of blank NPs (NPB) and nHA. As shown in Figure 2C and Figures 3A–F, at all concentrations of NPB and nHA, 293T cells showed high cell viability. CCK-8 assay showed similar results as live/dead dyeing. Notably, under high nHA treatment, the cell viability showed a slight reduction, while the cytotoxic effect was weaken by PEG-nHA coating, indicating that the hybridization effectively reduced the cytotoxicity non-tumorigenic cells like 293T cells. Taken together, the results above suggested the good biocompatibility and low cytotoxicity of our hybrid nanoparticles *in vitro*.

### HA-PEG-nHA-ZOL NPs Showed Low Tissue Toxicity *in vivo*

**FIGURE 4:**
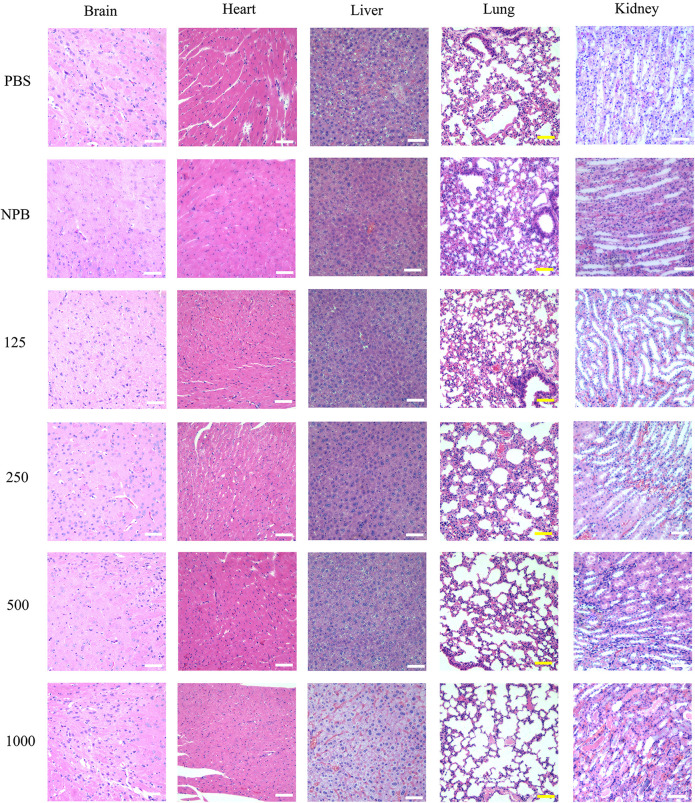
H&E staining of the brain, heart, liver, lung, and kidney. H&E staining images of the brain, heart, liver, lung, and kidney were obtained after mice were sacrificed at the 3rd day post the intravenous injection with 100 μl gradient concentration of NPs (125 μg/ml, 250 μg/ml, 500 μg/ml, 1,000 μg/ml), PBS and blank NPs (NPB). Scale bar: 50 μm.

H&E staining was performed to evaluate the *in vivo* toxicity of NPs at different concentrations. High-dose blank NPs (NPB, 1000 μg/ml) and PBS were used as control groups. Figure 4 shows the sections of the brain, heart, liver, lung, and kidney 3 days after drug treatment. When treated with NPB or PBS, all corresponding figures showed no tissue toxicity (Figure 4). For drug-loaded NP groups, when treated with low-dose NPs (<250 μg/ml), no obvious tissue toxicity was observed in all the sections. The brain, heart, liver, and lung did not show evident tissue toxicity under high-dose NP (>500 μg/ml) treatment, either (Figure 4). However, distinct cell death and tissue damage were observed in the kidney and liver when mice were treated with high-dose NPs (>500 μg/ml) (Figure 4). Especially when treated with 1000 μg/ml drug-loaded NPs, swollen renal tubules and liver tissue were observed. These results suggested the low *in vivo* tissue toxicity of HA-PEG-nHA-ZOL NPs in a proper dose range while tissue toxicity still existed under high-dose treatment.

### Low-Dose HA-PEG-nHA-ZOL NPs Had No Impact on Liver or Kidney Function

**FIGURE 5:**
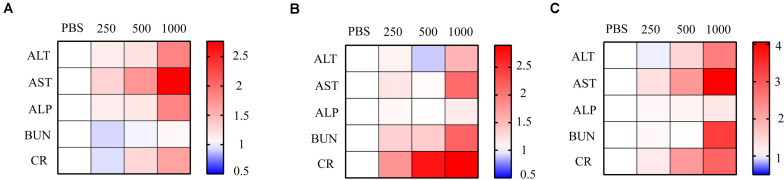
Relative ALT, AST, ALP, BUN, and CR level at 2, 3, and 5 days after NP injection. Relative levels of ALT, AST, ALP, BUN, and CR were presented as heat map. Gradient NPs (250 μg/ml, 500 μg/ml, 1,000 μg/ml) were injected into mice intravenously (*n* = 3). Liver and kidney function indicators were measured **(A)** 2 days, **(B)** 3 days, and **(C)** 5 days post NP treatment. PBS was used as the control *n* = 3.

Liver function was evaluated 2, 3, and 5 days after NP intravascular delivery according to serum ALT, AST, and ALP levels. A total of 2 days after NP injection, groups treated with 250 μg/ml NPs showed insignificant serum ALT and AST level variation (Figure 5A). However, the AST level had a significant increase under the 500-μg/ml NP treatment, while a more distinct increase in ALT, AST, and ALP levels was observed under the 1000-μg/ml NP treatment (Figure 5A). A total of 3 and 5 days after NP injection, the ALP value returned to normal level, while ALT and AST values were maintained at high levels, suggesting the possibility of long-term permanent liver injury or acute inflammation after high-dose NP treatment (Figures 5B,C).

On the other hand, BUN and CR levels were measured 2, 3, and 5 days after NP intravascular delivery to assess kidney functions. A total of 2 days after NP delivery, all groups had no significant variation in BUN and CR levels (Figure 5A). However, 3 and 5 days after NP injection, groups treated with low-dose NPs (<250 μg/ml) had no evident variation in BUN and CR levels, while high-dose (>500 μg/ml) NP injection led to a significant increase in BUN and CR levels, suggesting severe kidney dysfunction (Figures 5B,C).

In general, an appropriate amount of NP administration had no impact on liver and kidney function. However, high-dose NPs resulted in liver and kidney malfunction, which might be due to NP or NPB accumulation in glomerulus or renal tubules, as well as the high hepatotoxicity of circulating ZOL.

### Low-Dose HA-PEG-nHA-ZOL Had No Impact on Immune Cell Infiltration

**FIGURE 6:**
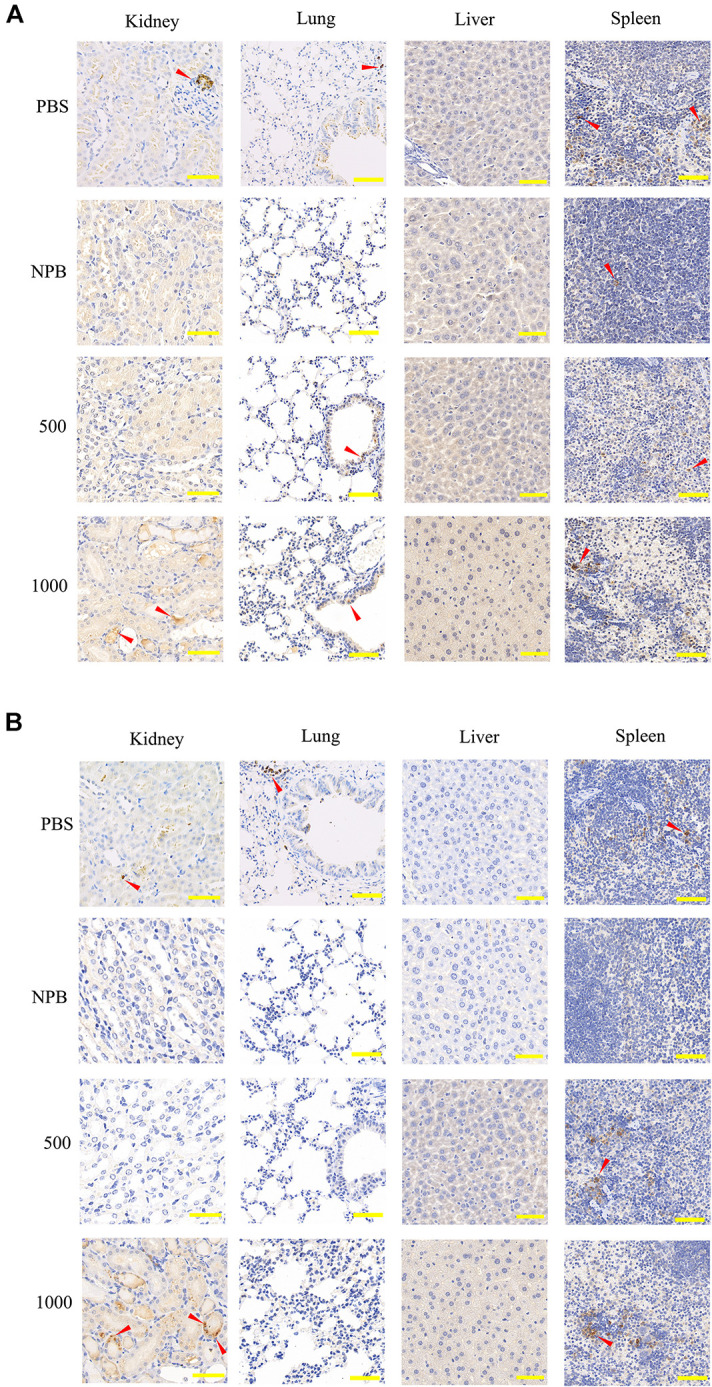
IHC staining of CD3 and CD68 in the kidney, lung, liver, and spleen. **(A)** CD3 and **(B)** CD68 IHC staining sections of the kidney, lung, liver, and spleen 3 days after high-dose NP injection (500 μg/ml, 1000 μg/ml). PBS and blank NPs (NPB) were used as the control group. Scale bar: kidney, 100 μm; lung, 200 μm; liver, 50 μm; spleen, 100 μm. Red arrow, infiltrated immune cells.

Gradient concentrations of NPs were injected via intravenous route (details in Table 3), and immunohistochemical (IHC) staining was performed 3 days after injection in order to investigate the tissue immune cell infiltration in the kidney, lung, liver, and spleen. High-dose blank NPs (NPB, 1000 μg/ml) and PBS were used as control groups. As shown in Figure 6, even with high-dose NP (>500 μg/ml) treatment, no apparent inflammation was observed in the lung, liver, and spleen. For kidney, with the 500-μg/ml NP treatment, CD3 cell (T lymphocyte) and CD68 cell (monocyte/macrophage) infiltration rates had no obvious increase, while with the 1000-μg/ml NP treatment, a higher immune cell filtration rate was observed (Figures 6A,B), indicating the severe inflammation which might be due to accumulation of NPs or NPB in glomerulus and renal tubules as predicted before. However, no obvious inflammation was observed in the liver, suggesting the possibility of liver injury due to high toxicity of circulating zoledronic acid.

### Low-Dose HA-PEG-nHA-ZOL Shows a Similar Immune Response Compared to ZOL

**FIGURE 7:**
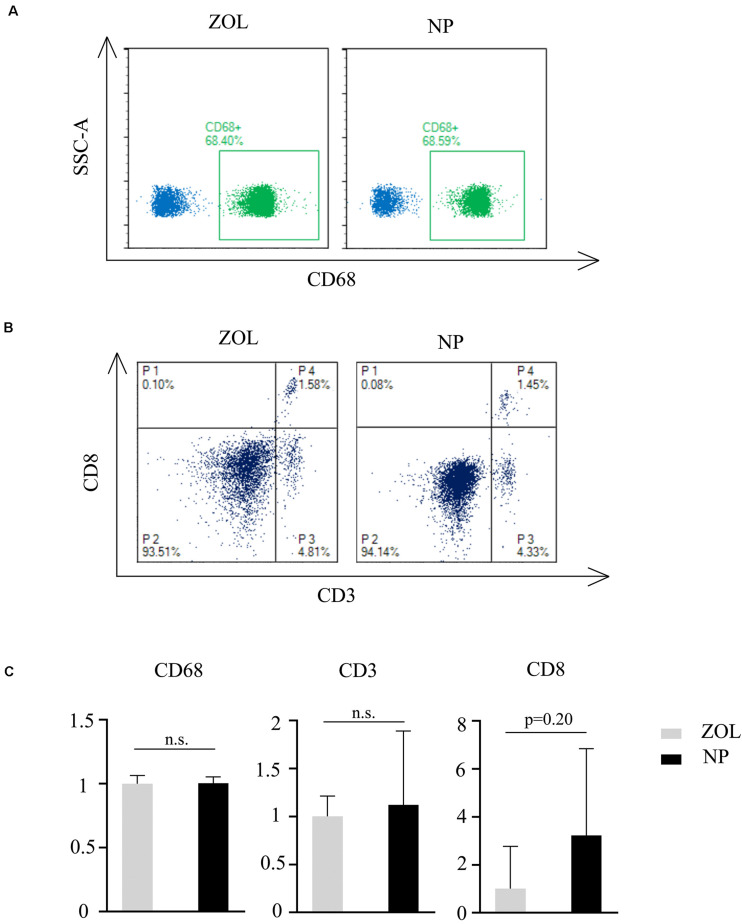
Flow cytometry analysis of peripheral blood. Flow cytometry classification of **(A)** CD68, **(B)** CD3, and CD8 cells after same-dose ZOL and NP treatment. P4, CD8 + /CD3 + lymphocytes; P3, CD8-/CD8 + cells. **(C)** Relative immune cell number after NP treatment compared to traditional ZOL treatment *n* = 4. Mann–Whitney *U* test, n.s., non-significance.

ZOL therapy showed a moderate immune response with low-dose treatment. To compare the immune response between classical ZOL therapy and hybrid NP therapy, 100 μl of 250 μg/ml NPs and 100 μg/ml ZOL were injected into mice in 2 groups (*n* = 4). Peripheral blood was collected, and flow cytometry analysis was performed 3 days post injection to measure the relative T cell and macrophage numbers. According to Figure 7A, CD68 + cell (monocytes/macrophages) number had no significant difference between the NP group and ZOL group. Meanwhile, CD3 + cell (T lymphocyte) and CD8 + cell (cytotoxic T lymphocyte) numbers showed no significant variation (Figure 7B). However, one mouse showed a strong immune response (two folds of T lymphocytes, eight folds of cytotoxic T lymphocytes) after NP injection (Figures 7B,C). The anomaly might be due to individual tolerance difference, which required further experiments with a larger sample size. In general, ZOL and NPs showed a similar and acceptable immune response after systemic delivery, which suggested that NPs could be exploited *in vivo* as a replacement of traditional ZOL therapy in the future.

## Discussion

In this study, we constructed a novel hybrid nanoparticle which combined the advantages of organic and inorganic material. The HA-PEG organic shell could achieve sustained drug release while HA-CD44 binding and PEG acidic-sensitive shedding were proposed to realize osteosarcoma-specific targeting and drug release.

Nano-hydroxyapatite, a widely applied inorganic biomedical material, showed good biocompatibility in bone and tooth repair. The nanoparticle we fabricated was rod-like in shape, based on the rod-shaped nHA core. The rod-like nHA was considered as the product of traditional synthetic routes, while recently, the new spheroid-shaped nHA could be synthesized by a green route (Aguilar et al., 2020). It was also reported that spheroid-shaped nHA might have a lower cytotoxicity and better biocompatibility (Aguilar et al., 2020). Hence, in future studies, it was quite an interesting aspect to study the effect of different shapes of the nanoparticles. In the drug loading field, previous studies showed that nano-hydroxyapatite could load antitumor drugs as a carrier (Dai et al., 2015). As the inorganic core, nHA had the potential to repair tumor-induced osteolysis directly. In our study, surprisingly, the nanoparticle had a high drug loading rate up to 40%. Previous study tried liposome encapsulation of zoledronic acid, which had a drug loading rate less than 10% (Shmeeda et al., 2013). Traditional ZOL-loaded polymer nanoparticles had a low loading rate as well. As previously reported, the ZOL loading rate of poly(lactic-co-glycolic acid) (PLGA) nanoparticles was only 1.4% (Li et al., 2019). However, nanoparticles using calcium hybridization have been shown to have a high drug load, which can reach about 50% or even higher (Au et al., 2016). The high drug loading rate in our nanoparticle was mainly due to the high affinity of bisphosphonates to hydroxyapatite according to a previous study, which recommended that the phosphate’s oxygen atoms in zoledronate could chelate the calcium ions in hydroxyapatite (Russell et al., 2008). The special P–C–P structure in bisphosphonate, which was called bone hook, also contributed to the high loading rate. The high affinity of the bone hook to calcium salts contributed to the bone targeting of bisphosphonates, which was widely used in the clinical field. For example, radioisotope-labeled bisphosphonates (e.g., ^99m^T_C_-HDP) can be used for bone radiography.

On the other hand, in the hybrid system, PEG also contributes to improving the encapsulation of zoledronate. PEG is a common drug carrier in the biomedical field. It can improve drug encapsulation and drug uptake or increase their deposition at tumor sites (Giger et al., 2013). Therefore, it was rational for fabricating these hybrid organic and inorganic microparticles to increase the drug loading efficiency.

The drug loading efficiency tested here is higher than that in the authors’ previous work. We used alendronate and hydroxyapatite only to fabricate chitosan microparticles and reached a drug loading efficiency of 22.218% (Wu et al., 2014). Other studies also showed that the hybrid incorporation could increase the drug loading efficiency (Jafari et al., 2016; Sarkar et al., 2018). Furthermore, hyaluronic acid acts as a ligand to target tumor cells via CD44, which enables the hybrid particles to target solid tumors with highly expressed CD44 on the cell surface. Taken together, all these elements combined in the hybrid system were performed to enhance their drug loading efficacy and tumor toxicity capability in their synergistic way.

From the *in vitro* cytotoxicity test results, we observed that zoledronate alone exhibited higher cytotoxicity than the nanoparticles loaded with zoledronate. The previous study also showed that teriflunomide- (TEF) and methotrexate- (MTX) loaded hydroxyapatite nanoparticles could reduce the drug dosage and side effects (Pandey et al., 2018). This is consistent with our *in vitro* results. Furthermore, the pure nHA particles or the blank hybrid particles alone did not affect the cell viability at all concentrations. This result demonstrated that the blank hybrid particles itself was biocompatible. Importantly, after hybrid with PEG-HA, the cytotoxic effect of nHA particles was weakened by PEG-HA coating, indicating that the hybridization effectively reduced the cytotoxicity to normal cells. Other studies also demonstrated that the PEG-HA system could diminish the unfavorable non-specific reactions in biological environment (Xu et al., 2017; Yan et al., 2019).

To evaluate the safety of *in vivo* use, we examine the visceral toxicity, main organ immune cell infiltration, liver and kidney function, and immune response after intravenous administration of NPs. All results exhibited slight tissue toxicity and immune response under NP treatment with proper tested doses. However, strong tissue toxicity and severe immune response were observed in the kidney after high-dose (1000 μg/ml) NP treatment. We predicted that it may be due to the aggregation and accumulation of unabsorbed NPB or nHA in the kidney. As previously mentioned, both PEG and HA are well-known biocompatible biomaterials and had been widely used in the biomedical field (Faust et al., 2018; Scala et al., 2018). PEG has been used in clinic as a drug carrier to improve drug loading and prolong drug delivery (Yan et al., 2019). However, nano-hydroxyapatite was reported with vascular toxicity (Santos et al., 2018). This is mainly due to its non-degradability in circulation and accumulation in the kidney. The conjecture provided future research direction in nanoparticle circulation and excretion, as well as long-term nanoparticle treatment in a low-dose manner (<250 μg/ml). As we mentioned in the introduction section, most previous studies only investigated nanoparticles’ cytotoxicity to tumor cells while our study systematically investigates its safety both *in vitro* and *in vivo*. Tissue damage, immune cell infiltration, and immune response were identified. Data showed that low-dose nanoparticles had a similar tissue reaction regarding the lymphocyte and macrophage infiltration compared with the control group. However, a high dose of nanoparticles evidently exhibited toxicity in mice. Pathological changes such as swelling could be seen in the organ slices, especially in the kidney. Moreover, the liver and kidney function test supported the change in each group. Even in the low-dose group, the mice experienced transient liver and kidney function fluctuation. However, most of them returned to normal after 3–5 days, which means the influence was reversible. Therefore, we performed the FACS of blood samples in mice to analyze the immune response at the low dose of intravenous injection. According to flow cytometry analysis results, there was no significant variation in immune response between traditional ZOL therapy and NP treatment, which was consistent with the findings in tissue H&E and IHC staining. Therefore, our results revealed that the hybrid nanoparticles might be useful for biomedical application.

However, in this study, we used normal epithelial cell lines and healthy animal models, which could not directly show the therapeutic efficiency of the new hybrid NPs. Hence, the specific cytotoxicity for osteosarcoma cell lines should be further studied. Our preliminary data revealed that NPs showed enhanced cytotoxicity for 143b cells (an osteosarcoma cell line) compared to zoledronic acid (data show in future publication), which simply confirmed the accurate targeting and higher efficiency of NPs. More *in vivo* studies such as intratumoral injection will also be performed in future research.

Interestingly, according to recent studies, the metastasis inhibition effect of zoledronic acid was controversial. Under similar concentrations of ZOL treatment, different studies showed different pulmonary metastasis inhibition effects. With 0.12 mg/kg, twice per weekly ZOL injection, Dass and Choong (2007) observed metastasis inhibition. With 0.1 mg/kg, twice per weekly ZOL injection, Wolfe et al. (2011) found no effect on metastasis, while Labrinidis et al. (2009) even observed metastasis promotion. The difference could be related to the slight difference in concentrations. However, as introduced before, the rapid clearance of ZOL results in the low effective concentration. Hence, the sustained drug release could significantly increase the ZOL effective concentration at the tumor site, leading to more efficient osteosarcoma therapy. Moreover, as more and more ZOL combination therapy was raised to achieve better therapeutic effects, we developed new ideals of constructing multidrug loading nanoparticles based on the existing hybrid NP, which defines our future research.

## Conclusion

In this study, the authors synthesized an organic–inorganic hybrid nanoparticle which consisted of an HA-PEG polymer shell and an nHA core. The hybrid nanoparticle realized the good biocompatibility, higher zoledronic acid loading efficiency, and sustained drug release. The HA-mediated targeting and PEG-mediated acid-sensitive drug release were proposed to achieve specific drug activity in osteosarcoma. The nHA contributed to the higher zoledronic acid loading rate while the tissue toxicity was offset by the HA-PEG organic shell. *In vitro* cytotoxicity test and *in vivo* tissue toxicity assay further confirmed the low cytotoxicity and visceral toxicity under low-dose nanoparticle treatment. Compared to traditional ZOL therapy, no severe immune response was observed, recommending the possibility that the new hybrid nanoparticle could be applied in future osteosarcoma therapy.

## Data Availability Statement

The original contributions presented in the study are included in the article/Supplementary Material, further inquiries can be directed to the corresponding author.

## Ethics Statement

The animal study was reviewed and approved by the Institutional Animal Care and Use Committee (IACUC) of Hunan Cancer Hospital (KYJJ-2016-009).

## Author Contributions

YX performed H&E staining, IHC staining, and liver and kidney function evaluation. ZZ performed live/dead dyeing, CCK-8 assay, and flow cytometry analysis. WZ fabricated the nanoparticle and performed characterization. WS performed H&E staining. ZZ, HW, and HW wrote the manuscript. CS and HW revised the manuscript. HW conceived all experiments. All authors contributed to the article and approved the submitted version.

## Conflict of Interest

The authors declare that the research was conducted in the absence of any commercial or financial relationships that could be construed as a potential conflict of interest.

## Supplementary Material

Supplementary Table 1 CCK-8 raw data.

Supplementary Table 2 Biochemical indexes raw data.

Supplementary Datasheet 1 Nanoparticle characteristics raw data.

Supplementary Datasheet 2 Flow cytometry analysis raw data.
