# Supplementary figures and images for: Zoledronic Acid-Loaded Hybrid Hyaluronic Acid/Polyethylene Glycol/Nano-Hydroxyapatite Nanoparticle: Novel Fabrication and Safety Verification
Source: Front Bioeng Biotechnol. 2021 Feb 15;9:629928. doi: 10.3389/fbioe.2021.629928 (PMC7917242; doi:10.3389/fbioe.2021.629928)

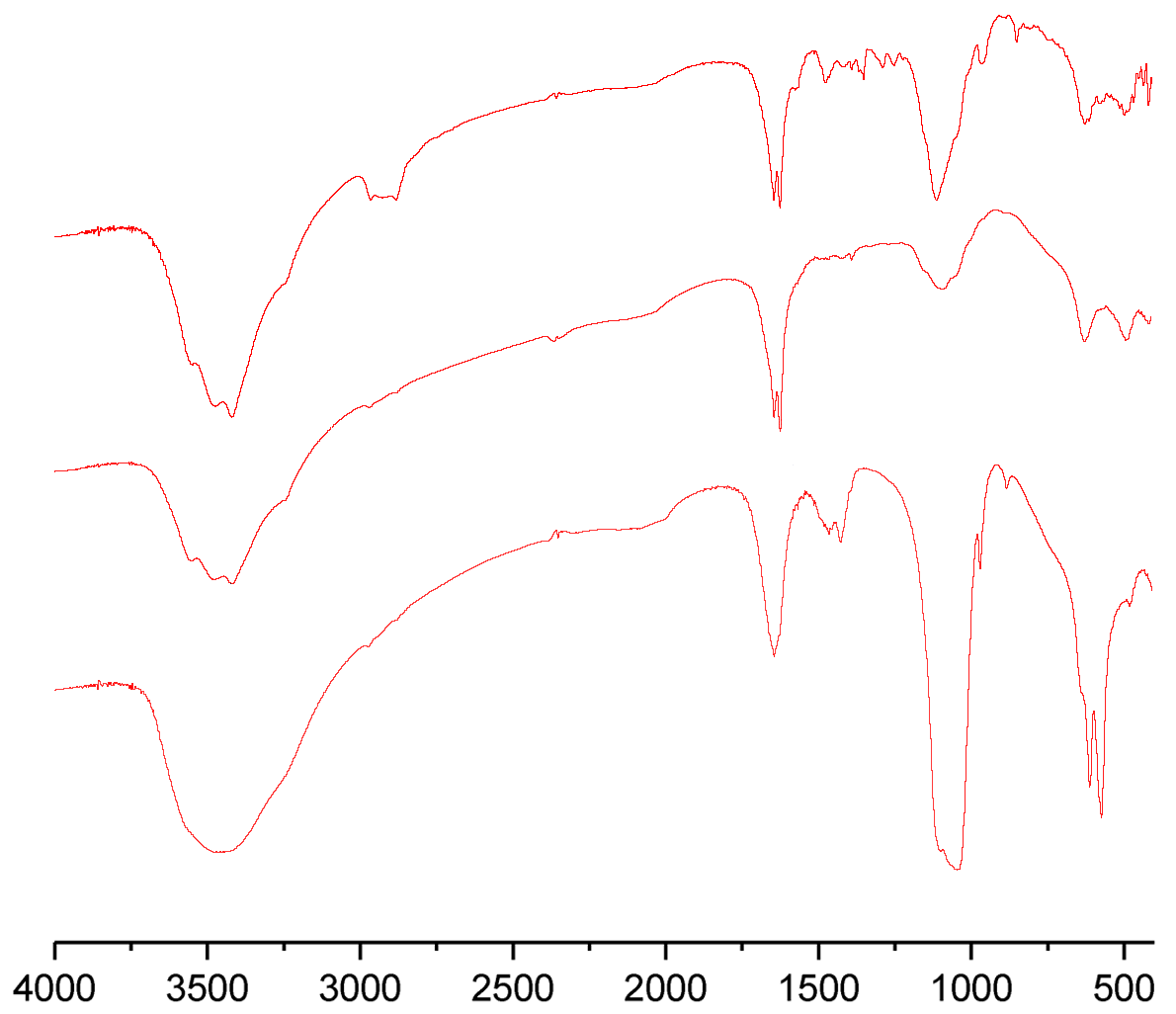

Supplement: Supplementary Datasheet 1 — Nanoparticle characteristics raw data. [file Data_Sheet_1.ZIP › Figure.1 Characteristics/FIRT.tif]

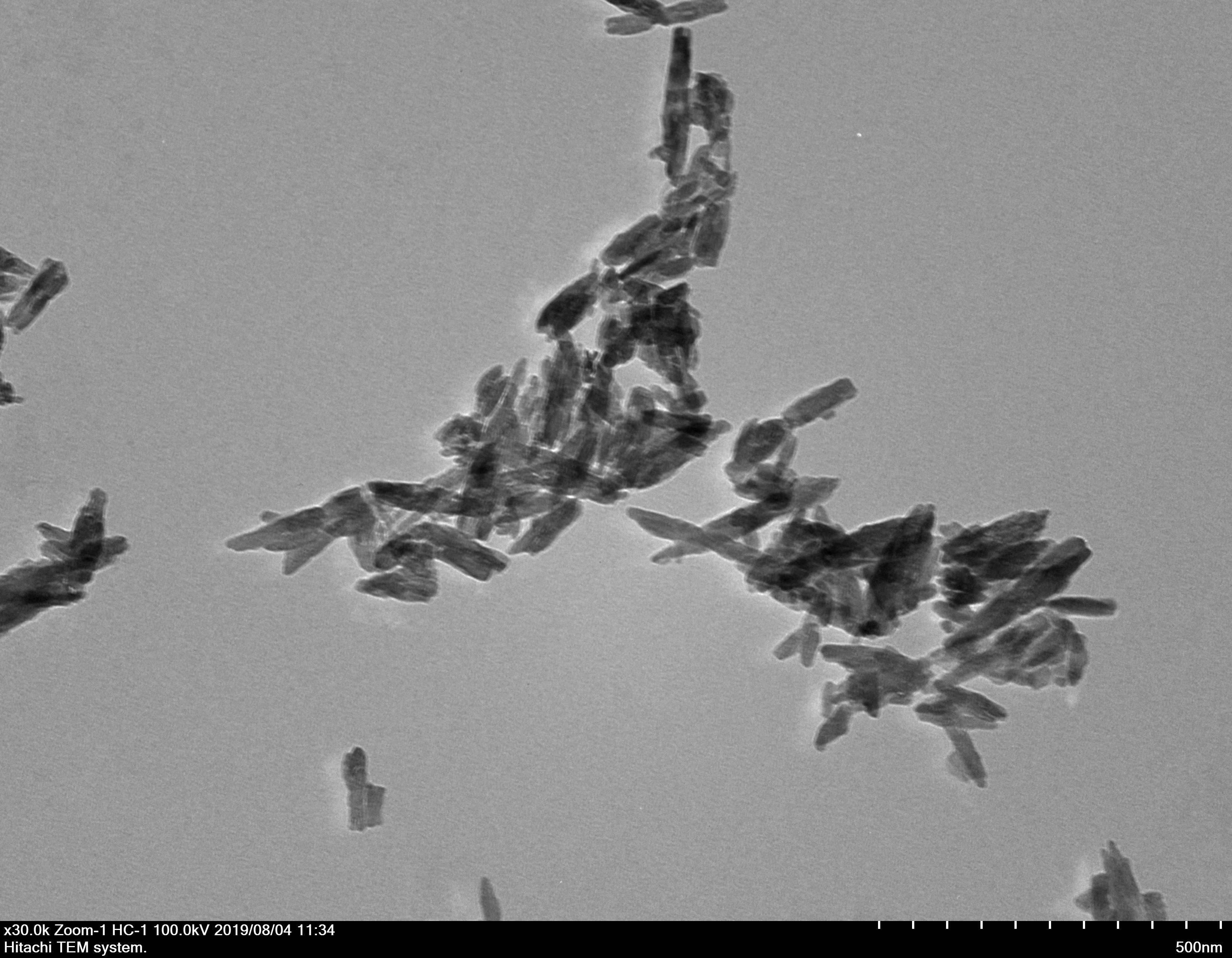

Supplement: Supplementary Datasheet 1 — Nanoparticle characteristics raw data. [file Data_Sheet_1.ZIP › Figure.1 Characteristics/NP SEM/1-1 HA-PEG-nHAú¿-ú⌐ZOL.tif]

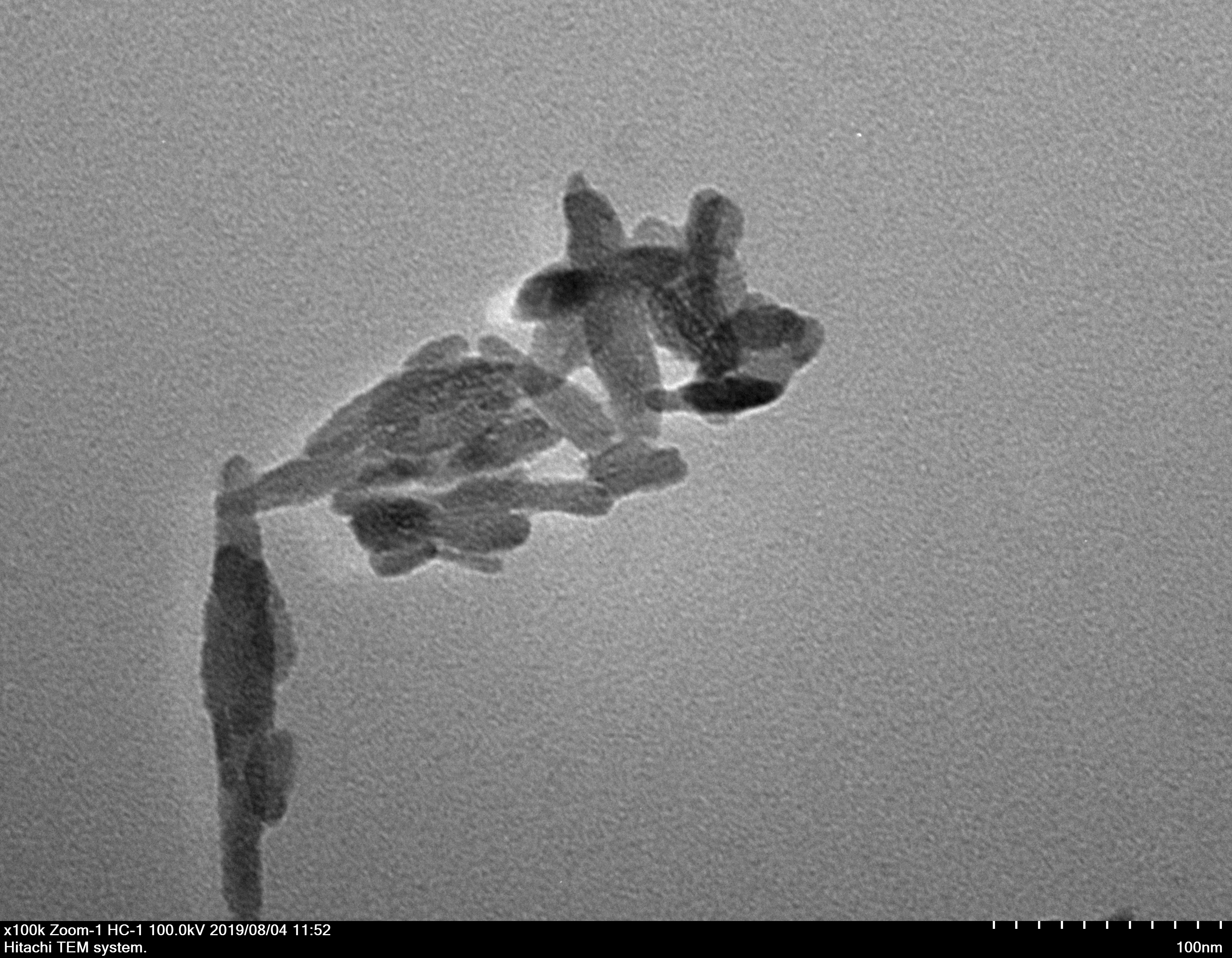

Supplement: Supplementary Datasheet 1 — Nanoparticle characteristics raw data. [file Data_Sheet_1.ZIP › Figure.1 Characteristics/NP SEM/2-2HA-PEG-nHA blank.tif]
