# Supplementary material for: Zoledronic Acid-Loaded Hybrid Hyaluronic Acid/Polyethylene Glycol/Nano-Hydroxyapatite Nanoparticle: Novel Fabrication and Safety Verification
Source: Front Bioeng Biotechnol. 2021 Feb 15;9:629928. doi: 10.3389/fbioe.2021.629928 (PMC7917242; doi:10.3389/fbioe.2021.629928)
Supplement: Supplementary Datasheet 2 — Flow cytometry analysis raw data. [file Data_Sheet_2.PDF]

Flow cytometry analysis report

Sample name: ZOL and NP analysis time：2020/9/26 14:34

machine：NovoCyte 452180228888 software：NovoExpress 1.4.1

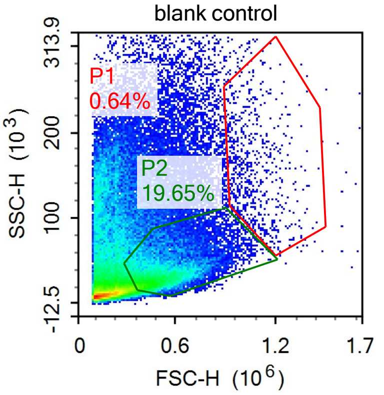

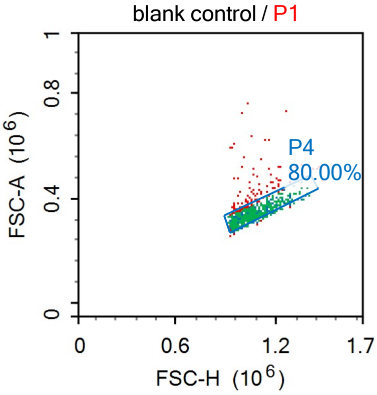

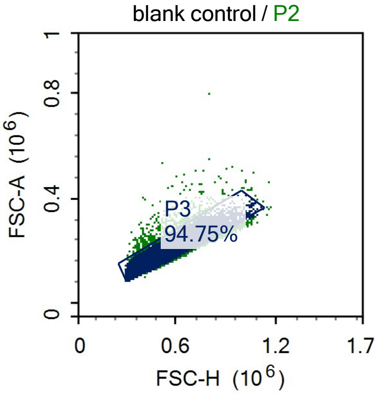

Gate	Count	% All	Median X	M
All	99,000	100.00%	241,687	2[illegible]
P1	630	0.64%	1,049,253	1[illegible]
P2	19,456	19.65%	484,930	34,559

Gate	Count	% P1	Median X	M
P1	630	100.00%	1,049,253	3[illegible]
P4	504	80.00%	1,050,614	3[illegible]

Gate	Count	% P2	Median X	Median Y
P2	19,456	100.00%	484,930	177,149
P3	18,435	94.75%	479,380	174,224

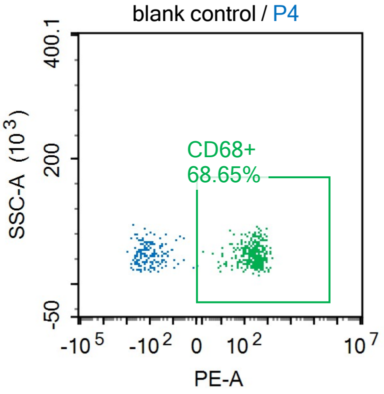

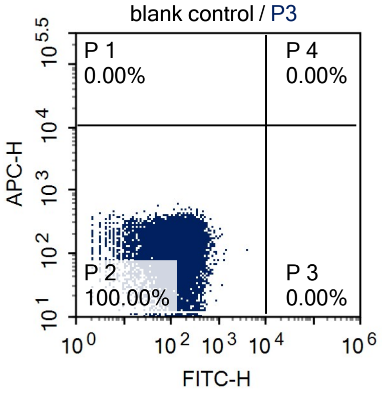

Gate	Count	% P4	Median X	M
P4	504	100.00%	157	4[illegible]
CD68+	346	68.65%	300	4[illegible]

Gate	Count	% P3	Median X	Median Y
P3	18,435	100.00%	108	103
P 1	0	0.00%	0	0
P 4	0	0.00%	0	0
P 2	18,435	100.00%	108	103
P 3	0	0.00%	0	0

样本统计表格 - blank control

Gate	Count	% Parent	X	Y	Median X	Median Y
All	99,000					
P1	630	0.64%	FSC-H	SSC-H	1,049,253	152,848
P4	504	80.00%	FSC-H	FSC-A	1,050,614	335,916
CD68+	346	68.65%	PE-A	SSC-A	300	47,301
P2	19,456	19.65%	FSC-H	SSC-H	484,930	34,559
P3	18,435	94.75%	FSC-H	FSC-A	479,380	174,224
P 1	0	0.00%	FITC-H	APC-H	0	0
P 4	0	0.00%	FITC-H	APC-H	0	0
P 2	18,435	100.00%	FITC-H	APC-H	108	103
P 3	0	0.00%	FITC-H	APC-H	0	0

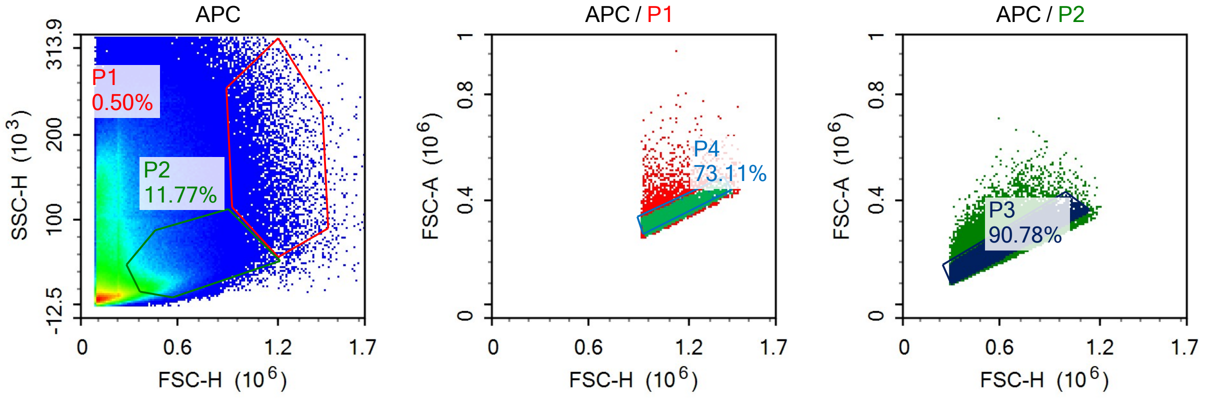

Gate	Count	% All	Median X	
All	1,129,744	100.00%	217,743	[illegible]
P1	5,600	0.50%	1,052,651	[illegible]
P2	133,015	11.77%	464,759	37,315

Gate	Count	% P1	Median X	M
P1	5,600	100.00%	1,052,651	34[illegible]
P4	4,094	73.11%	1,051,116	33[illegible]

Gate	Count	% P2	Median X	Median Y
P2	133,015	100.00%	464,759	177,161
P3	120,752	90.78%	458,644	172,517

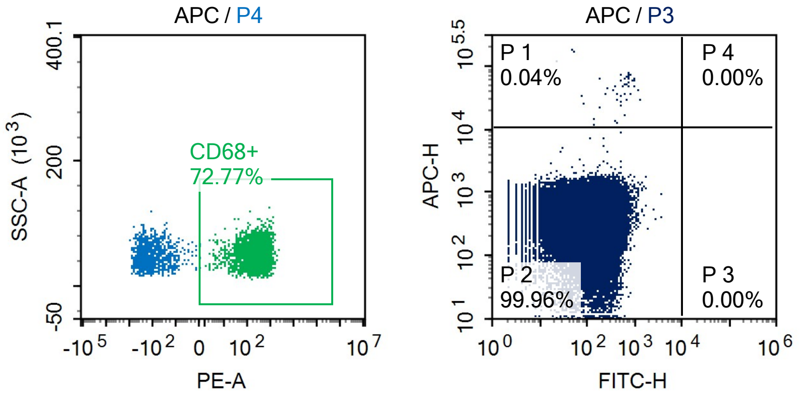

Gate	Count	% P4	Median X	M
P4	4,094	100.00%	191	4[illegible]
CD68+	2,979	72.77%	303	4[illegible]

Gate	Count	% P3	Median X	Median Y
P3	120,752	100.00%	111	446
P 1	50	0.04%	545	49,524
P 4	0	0.00%	0	0
P 2	120,702	99.96%	111	446
P 3	0	0.00%	0	0

样本统计表格 - APC

Gate	Count	% Parent	X	Y	Median X	Median Y
All	1,129,744					
P1	5,600	0.50%	FSC-H	SSC-H	1,052,651	156,929
P4	4,094	73.11%	FSC-H	FSC-A	1,051,116	336,865
CD68+	2,979	72.77%	PE-A	SSC-A	303	47,509
P2	133,015	11.77%	FSC-H	SSC-H	464,759	37,315
P3	120,752	90.78%	FSC-H	FSC-A	458,644	172,517
P 1	50	0.04%	FITC-H	APC-H	545	49,524
P 4	0	0.00%	FITC-H	APC-H	0	0
P 2	120,702	99.96%	FITC-H	APC-H	111	446
P 3	0	0.00%	FITC-H	APC-H	0	0

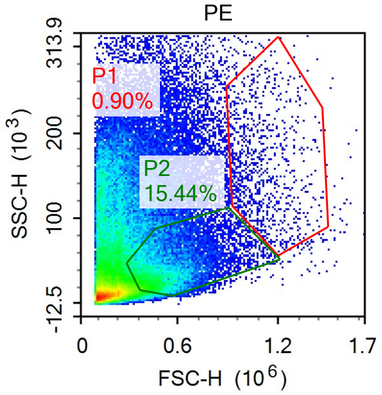

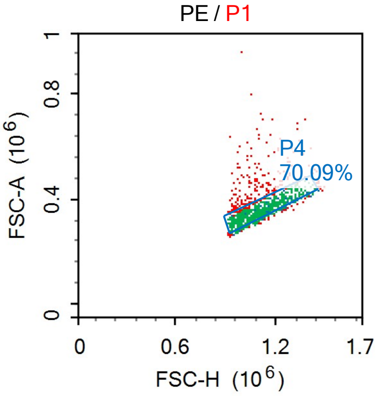

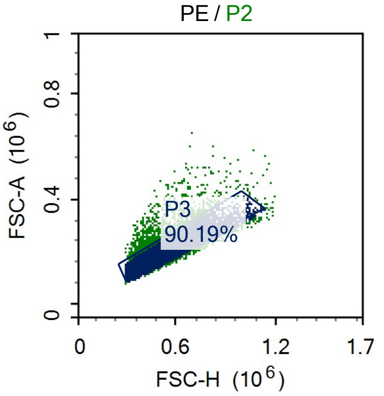

Gate	Count	% All	Median X	M
All	100,000	100.00%	235,076	3
P1	896	0.90%	1,085,682	1
P2	15,444	15.44%	480,058	37,438

Gate	Count	% P1	Median X	M
P1	896	100.00%	1,085,682	36
P4	628	70.09%	1,087,664	34

Gate	Count	% P2	Median X	Median Y
P2	15,444	100.00%	480,058	191,479
P3	13,929	90.19%	474,055	185,449

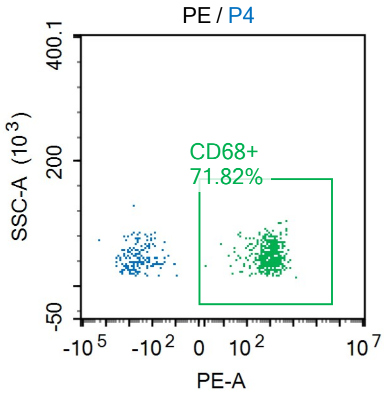

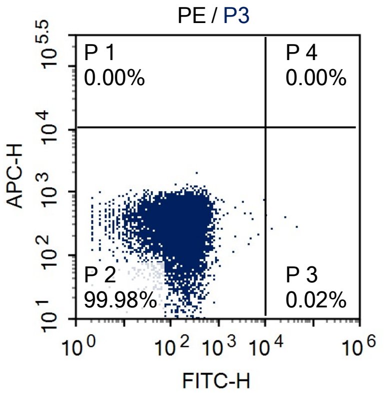

Gate	Count	% P4	Median X	M
P4	628	100.00%	613	45
CD68+	451	71.82%	1,147	46

Gate	Count	% P3	Median X	Median Y
P3	13,929	100.00%	156	306
P 1	0	0.00%	0	0
P 4	0	0.00%	0	0
P 2	13,926	99.98%	156	306
P 3	3	0.02%	25,927	273

样本统计表格 - PE

Gate	Count	% Parent	X	Y	Median X	Median Y
All	100,000					
P1	896	0.90%	FSC-H	SSC-H	1,085,682	151,808
P4	628	70.09%	FSC-H	FSC-A	1,087,664	346,888
CD68+	451	71.82%	PE-A	SSC-A	1,147	46,308
P2	15,444	15.44%	FSC-H	SSC-H	480,058	37,438
P3	13,929	90.19%	FSC-H	FSC-A	474,055	185,449
P 1	0	0.00%	FITC-H	APC-H	0	0
P 4	0	0.00%	FITC-H	APC-H	0	0
P 2	13,926	99.98%	FITC-H	APC-H	156	306
P 3	3	0.02%	FITC-H	APC-H	25,927	273

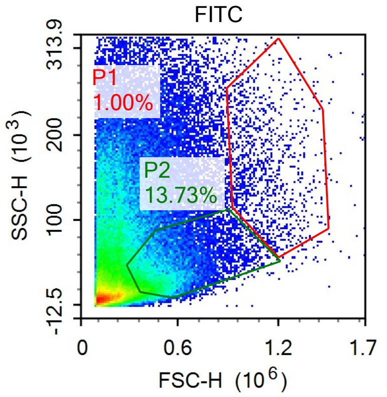

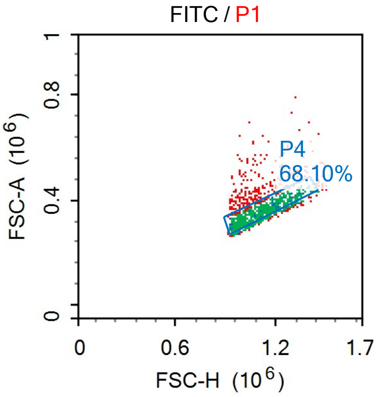

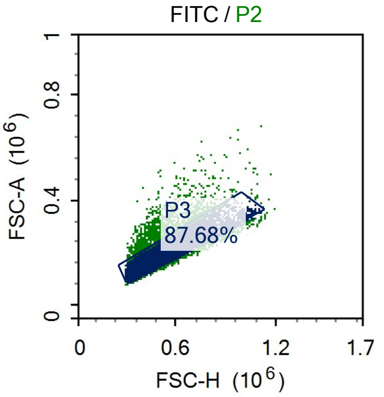

Gate	Count	% All	Median X	M[illegible]
All	100,000	100.00%	233,375	3[illegible]
P1	1,003	1.00%	1,090,353	1[illegible]
P2	13,730	13.73%	481,730	38,772

Gate	Count	% P1	Median X	M[illegible]
P1	1,003	100.00%	1,090,353	3[illegible]
P4	683	68.10%	1,091,603	3[illegible]

Gate	Count	% P2	Median X	Median Y
P2	13,730	100.00%	481,730	192,515
P3	12,039	87.68%	473,679	184,289

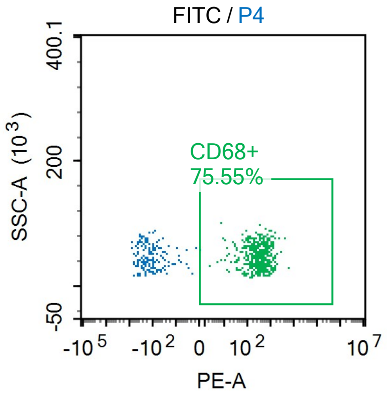

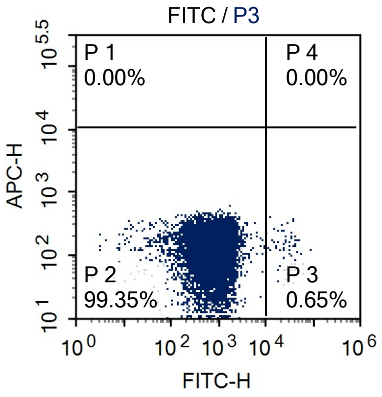

Gate	Count	% P4	Median X	M[illegible]
P4	683	100.00%	241	4[illegible]
CD68+	516	75.55%	366	4[illegible]

Gate	Count	% P3	Median X	Median Y
P3	12,039	100.00%	762	115
P 1	0	0.00%	0	0
P 4	0	0.00%	0	0
P 2	11,961	99.35%	757	115
P 3	78	0.65%	29,415	119

样本统计表格 - FITC

Gate	Count	% Parent	X	Y	Median X	Median Y
All	100,000					
P1	1,003	1.00%	FSC-H	SSC-H	1,090,353	155,705
P4	683	68.10%	FSC-H	FSC-A	1,091,603	347,382
CD68+	516	75.55%	PE-A	SSC-A	366	47,276
P2	13,730	13.73%	FSC-H	SSC-H	481,730	38,772
P3	12,039	87.68%	FSC-H	FSC-A	473,679	184,289
P 1	0	0.00%	FITC-H	APC-H	0	0
P 4	0	0.00%	FITC-H	APC-H	0	0
P 2	11,961	99.35%	FITC-H	APC-H	757	115
P 3	78	0.65%	FITC-H	APC-H	29,415	119

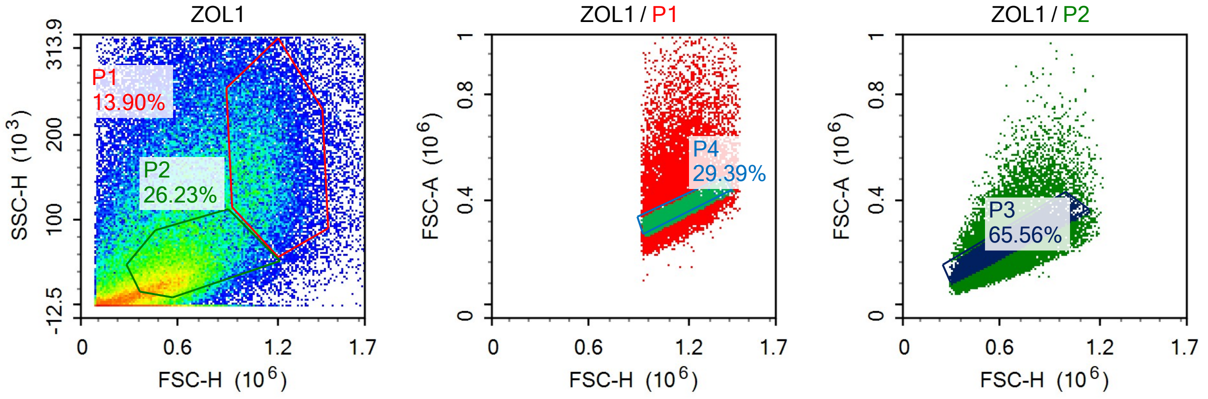

Gate	Count	% All	Median X	M
All	100,000	100.00%	580,639	8
P1	13,898	13.90%	1,118,203	1
P2	26,232	26.23%	588,682	42,943

Gate	Count	% P1	Median X	M
P1	13,898	100.00%	1,118,203	3
P4	4,085	29.39%	1,087,108	3

Gate	Count	% P2	Median X	Median Y
P2	26,232	100.00%	588,682	206,691
P3	17,199	65.56%	553,156	198,930

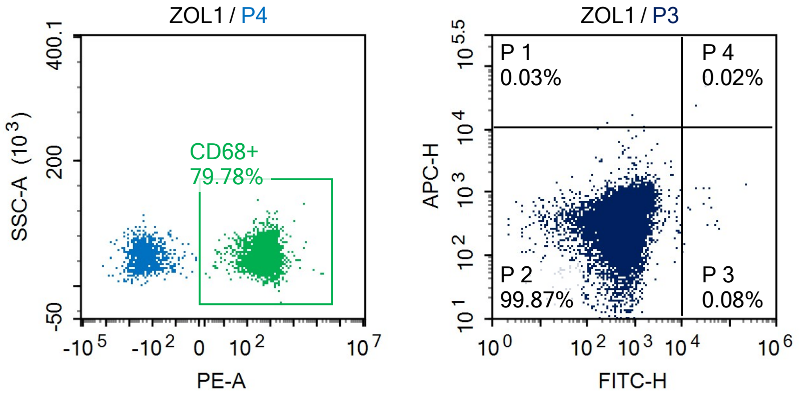

Gate	Count	% P4	Median X	M
P4	4,085	100.00%	520	4(
CD68+	3,259	79.78%	689	4(

Gate	Count	% P3	Median X	Median Y
P3	17,199	100.00%	532	321
P 1	5	0.03%	252	12,965
P 4	3	0.02%	30,810	48,465
P 2	17,177	99.87%	532	320
P 3	14	0.08%	33,942	581

样本统计表格 - ZOL1

Gate	Count	% Parent	X	Y	Median X	Median Y
All	100,000					
P1	13,898	13.90%	FSC-H	SSC-H	1,118,203	166,690
P4	4,085	29.39%	FSC-H	FSC-A	1,087,108	337,676
CD68+	3,259	79.78%	PE-A	SSC-A	689	46,839
P2	26,232	26.23%	FSC-H	SSC-H	588,682	42,943
P3	17,199	65.56%	FSC-H	FSC-A	553,156	198,930
P 1	5	0.03%	FITC-H	APC-H	252	12,965
P 4	3	0.02%	FITC-H	APC-H	30,810	48,465
P 2	17,177	99.87%	FITC-H	APC-H	532	320
P 3	14	0.08%	FITC-H	APC-H	33,942	581

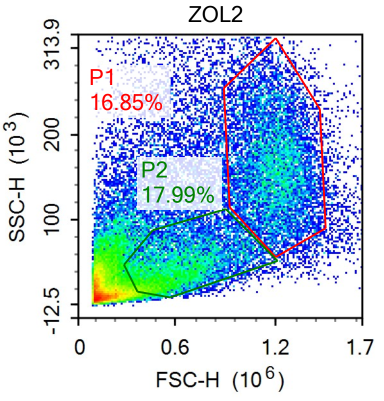

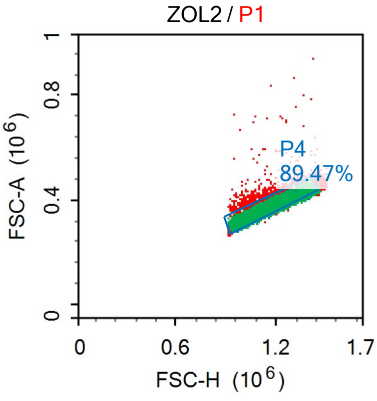

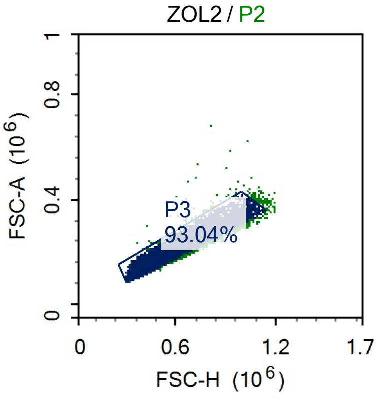

Gate	Count	% All	Median X	M	Gate	Count	% P1	Median X	M	Gate	Count	% P2	Median X	Median Y
All	48,810	100.00%	395,641	5[illegible]	P1	8,224	100.00%	1,192,449	3[illegible]	P2	8,783	100.00%	579,945	216,757
P1	8,224	16.85%	1,192,449	1[illegible]	P4	7,358	89.47%	1,193,728	3[illegible]	P3	8,172	93.04%	564,614	212,075
P2	8,783	17.99%	579,945	43,568										

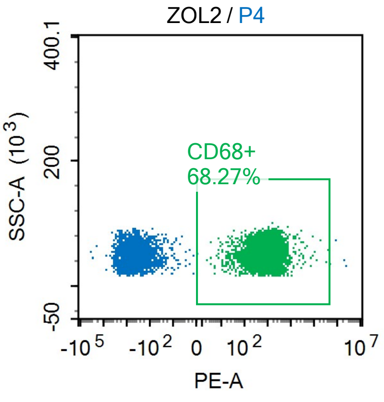

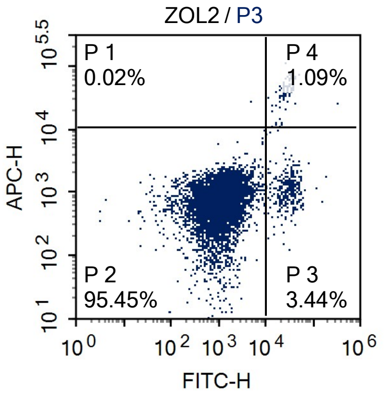

Gate	Count	% P4	Median X	M	Gate	Count	% P3	Median X	Median Y
P4	7,358	100.00%	581	4[illegible]	P3	8,172	100.00%	1,191	781
CD68+	5,023	68.27%	1,126	4[illegible]	P 1	2	0.02%	4,742	2,176,343
					P 4	89	1.09%	27,136	44,841
					P 2	7,800	95.45%	1,150	767
					P 3	281	3.44%	28,944	1,085

样本统计表格 - ZOL2

Gate	Count	% Parent	X	Y	Median X	Median Y
All	48,810					
P1	8,224	16.85%	FSC-H	SSC-H	1,192,449	161,750
P4	7,358	89.47%	FSC-H	FSC-A	1,193,728	375,109
CD68+	5,023	68.27%	PE-A	SSC-A	1,126	49,268
P2	8,783	17.99%	FSC-H	SSC-H	579,945	43,568
P3	8,172	93.04%	FSC-H	FSC-A	564,614	212,075
P 1	2	0.02%	FITC-H	APC-H	4,742	2,176,343
P 4	89	1.09%	FITC-H	APC-H	27,136	44,841
P 2	7,800	95.45%	FITC-H	APC-H	1,150	767
P 3	281	3.44%	FITC-H	APC-H	28,944	1,085

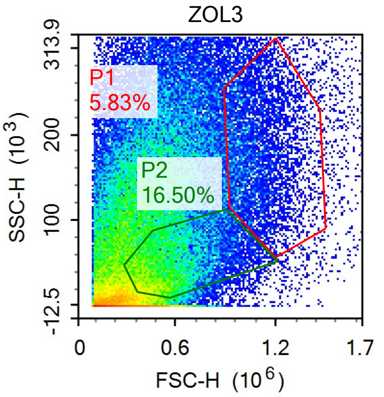

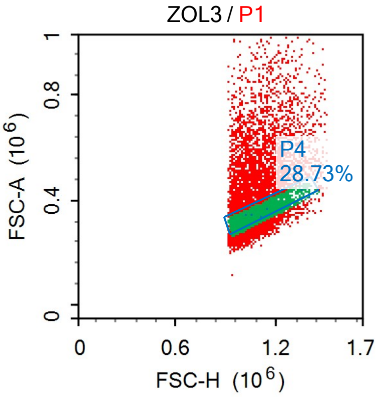

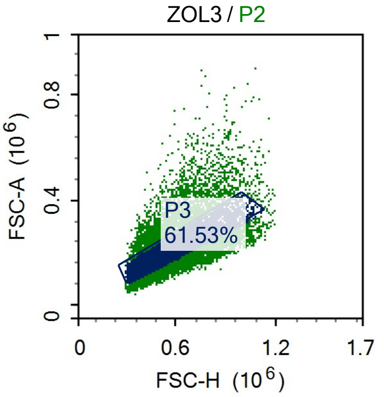

Gate	Count	% All	Median X	M
All	100,000	100.00%	421,135	8[illegible]
P1	5,827	5.83%	1,083,052	1[illegible]
P2	16,502	16.50%	543,589	47,375

Gate	Count	% P1	Median X	M
P1	5,827	100.00%	1,083,052	4[illegible]
P4	1,674	28.73%	1,071,815	3[illegible]

Gate	Count	% P2	Median X	Median Y
P2	16,502	100.00%	543,589	196,115
P3	10,153	61.53%	514,874	186,695

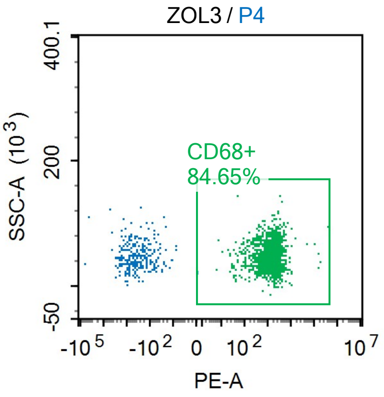

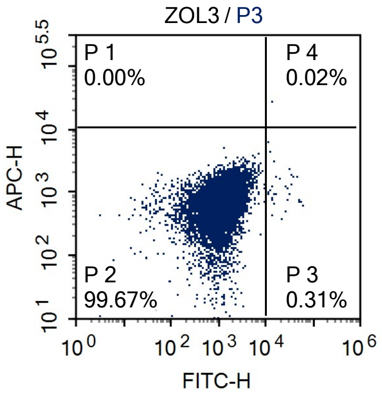

Gate	Count	% P4	Median X	M
P4	1,674	100.00%	1,101	4[illegible]
CD68+	1,417	84.65%	1,355	4[illegible]

Gate	Count	% P3	Median X	Median Y
P3	10,153	100.00%	1,120	646
P 1	0	0.00%	0	0
P 4	2	0.02%	19,693	47,046
P 2	10,120	99.67%	1,118	645
P 3	31	0.31%	20,421	1,002

样本统计表格 - ZOL3

Gate	Count	% Parent	X	Y	Median X	Median Y
All	100,000					
P1	5,827	5.83%	FSC-H	SSC-H	1,083,052	179,002
P4	1,674	28.73%	FSC-H	FSC-A	1,071,815	338,198
CD68+	1,417	84.65%	PE-A	SSC-A	1,355	48,212
P2	16,502	16.50%	FSC-H	SSC-H	543,589	47,375
P3	10,153	61.53%	FSC-H	FSC-A	514,874	186,695
P 1	0	0.00%	FITC-H	APC-H	0	0
P 4	2	0.02%	FITC-H	APC-H	19,693	47,046
P 2	10,120	99.67%	FITC-H	APC-H	1,118	645
P 3	31	0.31%	FITC-H	APC-H	20,421	1,002

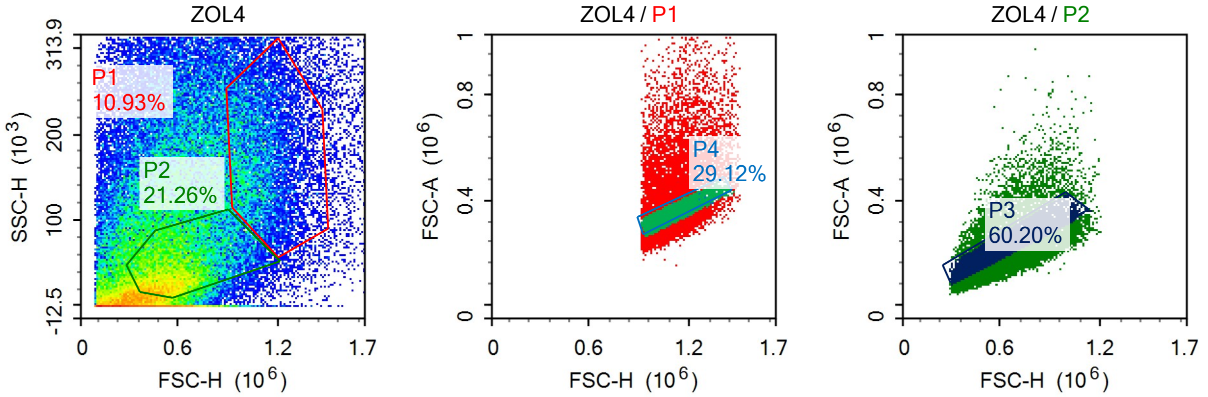

Gate	Count	% All	Median X	M	Gate	Count	% P1	Median X	M	Gate	Count	% P2	Median X	Median Y
All	100,000	100.00%	559,956	9	P1	10,932	100.00%	1,097,807	3	P2	21,262	100.00%	585,355	197,011
P1	10,932	10.93%	1,097,807	1	P4	3,183	29.12%	1,079,151	3	P3	12,799	60.20%	552,626	193,437
P2	21,262	21.26%	585,355	44,883										

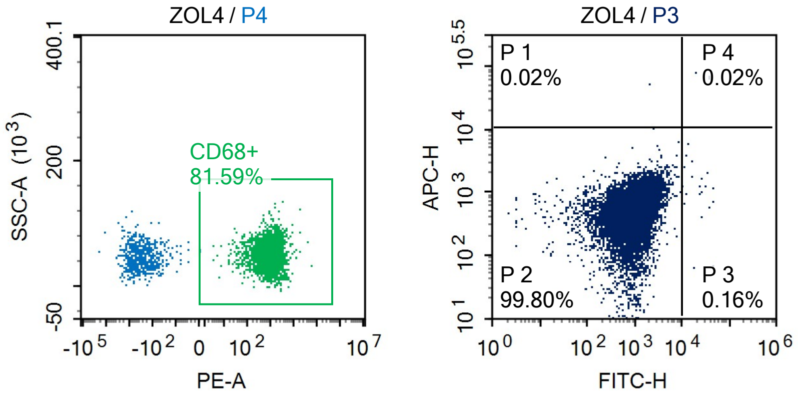

Gate	Count	% P4	Median X	M	Gate	Count	% P3	Median X	Median Y
P4	3,183	100.00%	782	4	P3	12,799	100.00%	899	501
CD68+	2,597	81.59%	989	4	P 1	2	0.02%	2,211	32,666
					P 4	3	0.02%	36,450	71,212
					P 2	12,774	99.80%	898	500
					P 3	20	0.16%	23,937	1,190

样本统计表格 - ZOL4

Gate	Count	% Parent	X	Y	Median X	Median Y
All	100,000					
P1	10,932	10.93%	FSC-H	SSC-H	1,097,807	173,416
P4	3,183	29.12%	FSC-H	FSC-A	1,079,151	336,599
CD68+	2,597	81.59%	PE-A	SSC-A	989	47,966
P2	21,262	21.26%	FSC-H	SSC-H	585,355	44,883
P3	12,799	60.20%	FSC-H	FSC-A	552,626	193,437
P 1	2	0.02%	FITC-H	APC-H	2,211	32,666
P 4	3	0.02%	FITC-H	APC-H	36,450	71,212
P 2	12,774	99.80%	FITC-H	APC-H	898	500
P 3	20	0.16%	FITC-H	APC-H	23,937	1,190

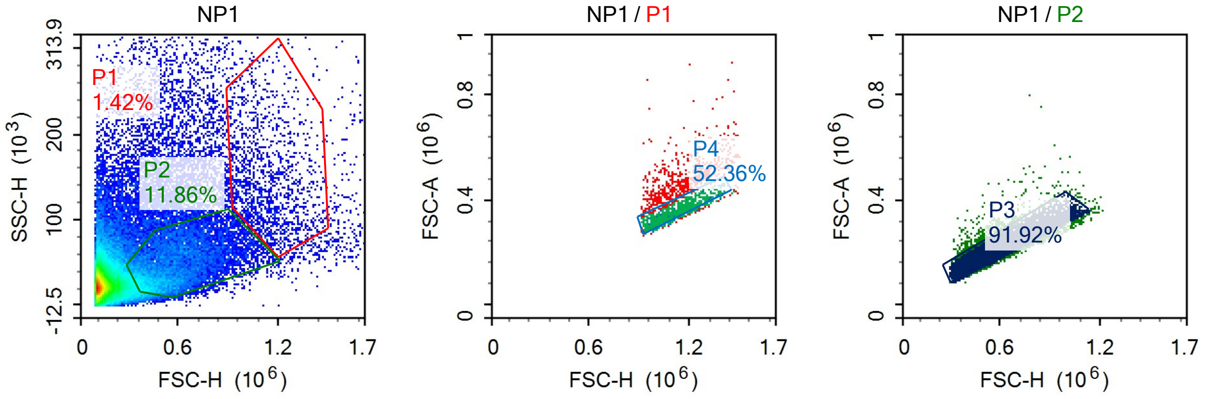

Gate	Count	% All	Median X	M	Gate	Count	% P1	Median X	M	Gate	Count	% P2	Median X	Median Y
All	100,000	100.00%	158,786	2[illegible]	P1	1,421	100.00%	1,130,099	3[illegible]	P2	11,863	100.00%	526,028	199,792
P1	1,421	1.42%	1,130,099	1[illegible]	P4	744	52.36%	1,107,049	3[illegible]	P3	10,905	91.92%	522,218	195,631
P2	11,863	11.86%	526,028	41,796										

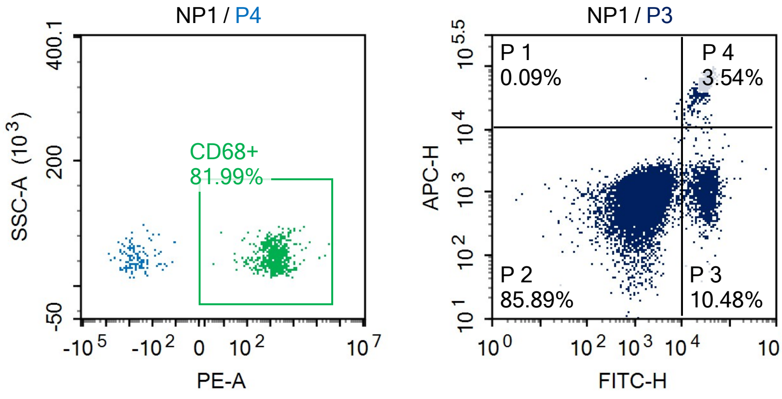

Gate	Count	% P4	Median X	M	Gate	Count	% P3	Median X	Median Y
P4	744	100.00%	1,253	4[illegible]	P3	10,905	100.00%	1,751	848
CD68+	610	81.99%	1,655	4[illegible]	P 1	10	0.09%	8,110	23,159
					P 4	386	3.54%	28,618	49,866
					P 2	9,366	85.89%	1,540	793
					P 3	1,143	10.48%	31,418	1,111

样本统计表格 - NP1

Gate	Count	% Parent	X	Y	Median X	Median Y
All	100,000					
P1	1,421	1.42%	FSC-H	SSC-H	1,130,099	133,570
P4	744	52.36%	FSC-H	FSC-A	1,107,049	351,706
CD68+	610	81.99%	PE-A	SSC-A	1,655	44,529
P2	11,863	11.86%	FSC-H	SSC-H	526,028	41,796
P3	10,905	91.92%	FSC-H	FSC-A	522,218	195,631
P 1	10	0.09%	FITC-H	APC-H	8,110	23,159
P 4	386	3.54%	FITC-H	APC-H	28,618	49,866
P 2	9,366	85.89%	FITC-H	APC-H	1,540	793
P 3	1,143	10.48%	FITC-H	APC-H	31,418	1,111

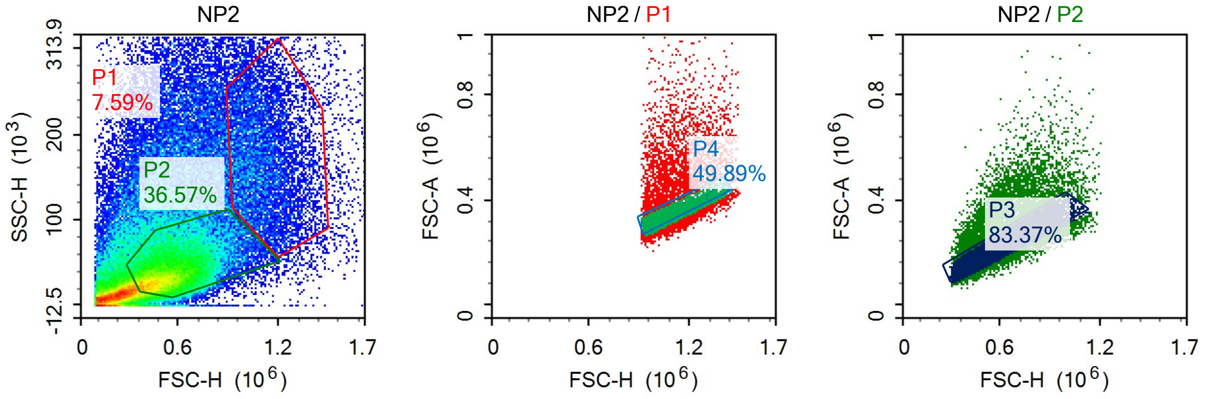

Gate	Count	% All	Median X	M
All	100,000	100.00%	467,458	4
P1	7,585	7.59%	1,108,507	1
P2	36,569	36.57%	538,351	38,250

Gate	Count	% P1	Median X	M
P1	7,585	100.00%	1,108,507	3
P4	3,784	49.89%	1,085,857	3

Gate	Count	% P2	Median X	Median Y
P2	36,569	100.00%	538,351	210,581
P3	30,487	83.37%	522,467	199,546

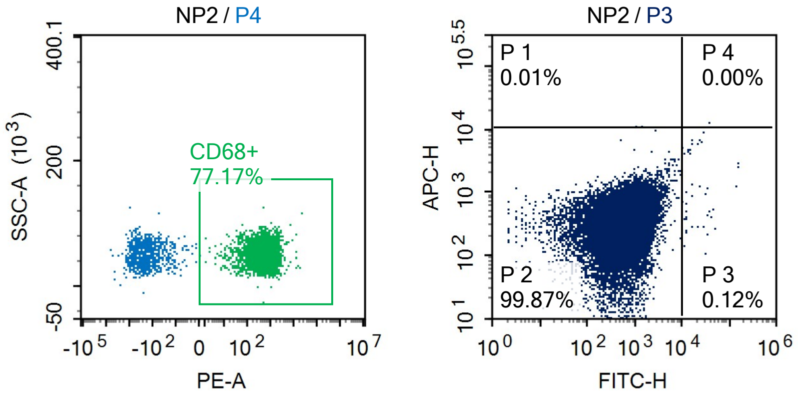

Gate	Count	% P4	Median X	M
P4	3,784	100.00%	398	5(
CD68+	2,920	77.17%	569	5(

Gate	Count	% P3	Median X	Median Y
P3	30,487	100.00%	599	357
P 1	2	0.01%	1,181	10,871
P 4	1	0.00%	37,421	12,931
P 2	30,446	99.87%	598	357
P 3	38	0.12%	18,780	899

样本统计表格 - NP2

Gate	Count	% Parent	X	Y	Median X	Median Y
All	100,000					
P1	7,585	7.59%	FSC-H	SSC-H	1,108,507	167,096
P4	3,784	49.89%	FSC-H	FSC-A	1,085,857	339,866
CD68+	2,920	77.17%	PE-A	SSC-A	569	50,254
P2	36,569	36.57%	FSC-H	SSC-H	538,351	38,250
P3	30,487	83.37%	FSC-H	FSC-A	522,467	199,546
P 1	2	0.01%	FITC-H	APC-H	1,181	10,871
P 4	1	0.00%	FITC-H	APC-H	37,421	12,931
P 2	30,446	99.87%	FITC-H	APC-H	598	357
P 3	38	0.12%	FITC-H	APC-H	18,780	899

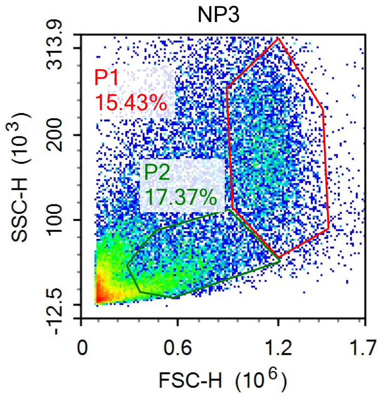

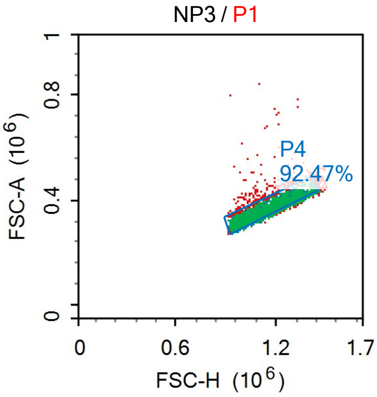

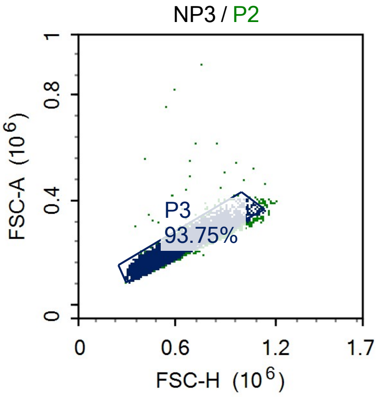

Gate	Count	% All	Median X	M
All	31,760	100.00%	457,399	6
P1	4,900	15.43%	1,130,375	1
P2	5,517	17.37%	574,636	42,335

Gate	Count	% P1	Median X	M
P1	4,900	100.00%	1,130,375	3
P4	4,531	92.47%	1,129,740	3

Gate	Count	% P2	Median X	Median Y
P2	5,517	100.00%	574,636	220,359
P3	5,172	93.75%	561,495	216,597

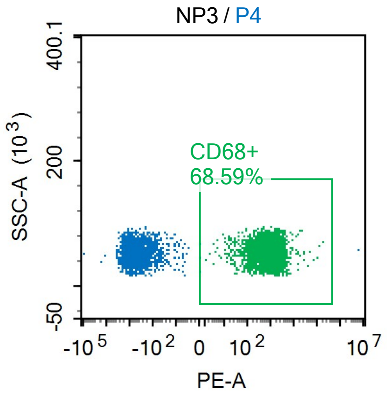

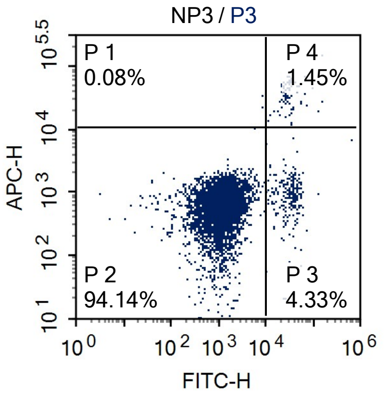

Gate	Count	% P4	Median X	M
P4	4,531	100.00%	465	5
CD68+	3,108	68.59%	931	5

Gate	Count	% P3	Median X	Median Y
P3	5,172	100.00%	1,261	657
P 1	4	0.08%	6,836	28,355
P 4	75	1.45%	29,599	37,506
P 2	4,869	94.14%	1,206	637
P 3	224	4.33%	33,417	913

样本统计表格 - NP3

Gate	Count	% Parent	X	Y	Median X	Median Y
All	31,760					
P1	4,900	15.43%	FSC-H	SSC-H	1,130,375	175,793
P4	4,531	92.47%	FSC-H	FSC-A	1,129,740	355,093
CD68+	3,108	68.59%	PE-A	SSC-A	931	52,887
P2	5,517	17.37%	FSC-H	SSC-H	574,636	42,335
P3	5,172	93.75%	FSC-H	FSC-A	561,495	216,597
P 1	4	0.08%	FITC-H	APC-H	6,836	28,355
P 4	75	1.45%	FITC-H	APC-H	29,599	37,506
P 2	4,869	94.14%	FITC-H	APC-H	1,206	637
P 3	224	4.33%	FITC-H	APC-H	33,417	913

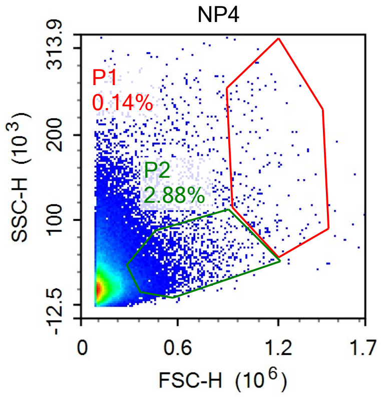

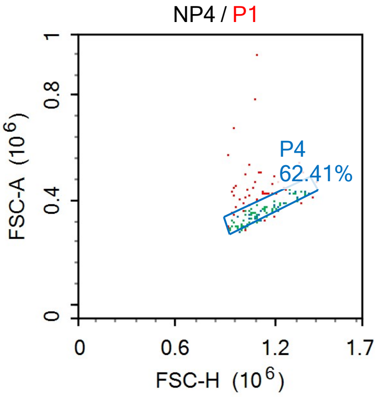

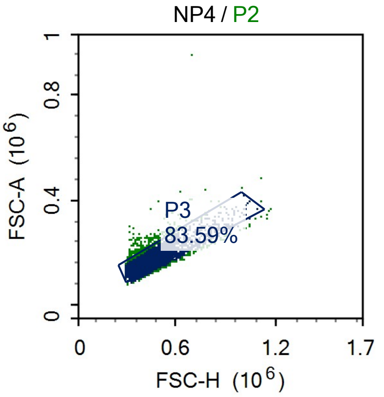

Gate	Count	% All	Median X	M
All	100,000	100.00%	141,743	2
P1	141	0.14%	1,081,397	1
P2	2,883	2.88%	415,032	39,762

Gate	Count	% P1	Median X	M
P1	141	100.00%	1,081,397	3(
P4	88	62.41%	1,080,838	34

Gate	Count	% P2	Median X	Median Y
P2	2,883	100.00%	415,032	169,121
P3	2,410	83.59%	407,711	162,255

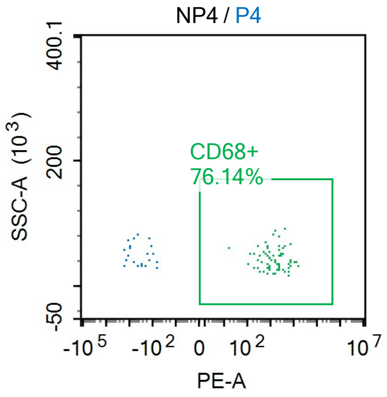

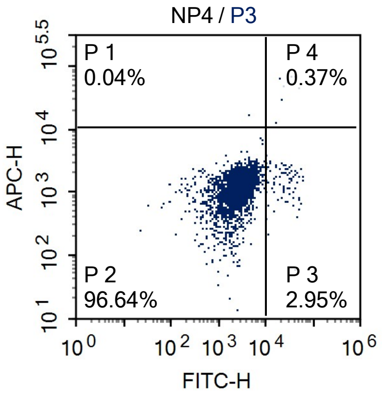

Gate	Count	% P4	Median X	M
P4	88	100.00%	820	4:
CD68+	67	76.14%	1,520	4'

Gate	Count	% P3	Median X	Median Y
P3	2,410	100.00%	2,433	1,097
P 1	1	0.04%	4,051	16,839
P 4	9	0.37%	26,900	51,761
P 2	2,329	96.64%	2,358	1,085
P 3	71	2.95%	26,245	1,542

样本统计表格 - NP4

Gate	Count	% Parent	X	Y	Median X	Median Y
All	100,000					
P1	141	0.14%	FSC-H	SSC-H	1,081,397	136,437
P4	88	62.41%	FSC-H	FSC-A	1,080,838	348,927
CD68+	67	76.14%	PE-A	SSC-A	1,520	41,819
P2	2,883	2.88%	FSC-H	SSC-H	415,032	39,762
P3	2,410	83.59%	FSC-H	FSC-A	407,711	162,255
P 1	1	0.04%	FITC-H	APC-H	4,051	16,839
P 4	9	0.37%	FITC-H	APC-H	26,900	51,761
P 2	2,329	96.64%	FITC-H	APC-H	2,358	1,085
P 3	71	2.95%	FITC-H	APC-H	26,245	1,542
